# Supplementation of Regular Diet With Medium-Chain Triglycerides for Procognitive Effects: A Narrative Review

**DOI:** 10.3389/fnut.2022.934497

**Published:** 2022-07-15

**Authors:** Ksenia Shcherbakova, Alexander Schwarz, Sergey Apryatin, Marina Karpenko, Alexander Trofimov

**Affiliations:** ^1^I.P. Pavlov Department of Physiology, Institute of Experimental Medicine, Saint Petersburg, Russia; ^2^Laboratory of the Molecular Mechanisms of Neuronal Interactions, Institute of Evolutionary Physiology and Biochemistry (RAS), Saint Petersburg, Russia

**Keywords:** ketosis, medium-chain fatty acids, medium-chain triglycerides, cardiometabolic health, neuroprotection, procognitive activity, caprylic acid (C8), capric acid (C10)

## Abstract

It is now widely accepted that ketosis (a physiological state characterized by elevated plasma ketone body levels) possesses a wide range of neuroprotective effects. There is a growing interest in the use of ketogenic supplements, including medium-chain triglycerides (MCT), to achieve intermittent ketosis without adhering to a strict ketogenic diet. MCT supplementation is an inexpensive and simple ketogenic intervention, proven to benefit both individuals with normal cognition and those suffering from mild cognitive impairment, Alzheimer's disease, and other cognitive disorders. The commonly accepted paradigm underlying MCT supplementation trials is that the benefits stem from ketogenesis and that MCT supplementation is safe. However, medium-chain fatty acids (MCFAs) may also exert effects in the brain directly. Moreover, MCFAs, long-chain fatty acids, and glucose participate in mutually intertwined metabolic pathways. Therefore, the metabolic effects must be considered if the desired procognitive effects require administering MCT in doses larger than 1 g/kg. This review summarizes currently available research on the procognitive effects of using MCTs as a supplement to regular feed/diet without concomitant reduction of carbohydrate intake and focuses on the revealed mechanisms linked to particular MCT metabolites (ketone bodies, MCFAs), highlighting open questions and potential considerations.

## Introduction

It is well established that ketogenic diet (KD) and ketone bodies (KB) exert many neuroprotective effects, such as providing an alternative energy source for the brain cells, modulating neurotransmission, supporting the antioxidant and anti-inflammatory responses. Several extensive reviews have been written on this topic (1–4). Although glucose is the primary energy source in the brain, cerebral glucose metabolism is often reduced in aged individuals and patients with Alzheimer's disease (AD), while the brain ketone body metabolism seems to remain intact (5–7). Therefore, providing the brain with ketone bodies in one way or another has emerged as an approach to support cognitive function—a concept that recently acquired a name: neuroketotherapeutics (8). Historically, the state of ketosis for the sake of harnessing its neuroprotective effects has been achieved through adhering to KD, long known as a treatment of intractable epilepsy (9, 10). Despite its efficacy, the use of KD is limited due to the severity of this dietary regime, which requires an almost complete reduction of dietary carbohydrates to enable ketogenesis from the long-chain fatty acids (LCFA) in the liver. A few alternative strategies have been proposed to achieve ketosis intermittently by adding various ketogenic supplements to a regular diet, including the medium-chain triglycerides (MCTs), as well as the salts and esters of ketone bodies (2, 11).

**Figure 1:**
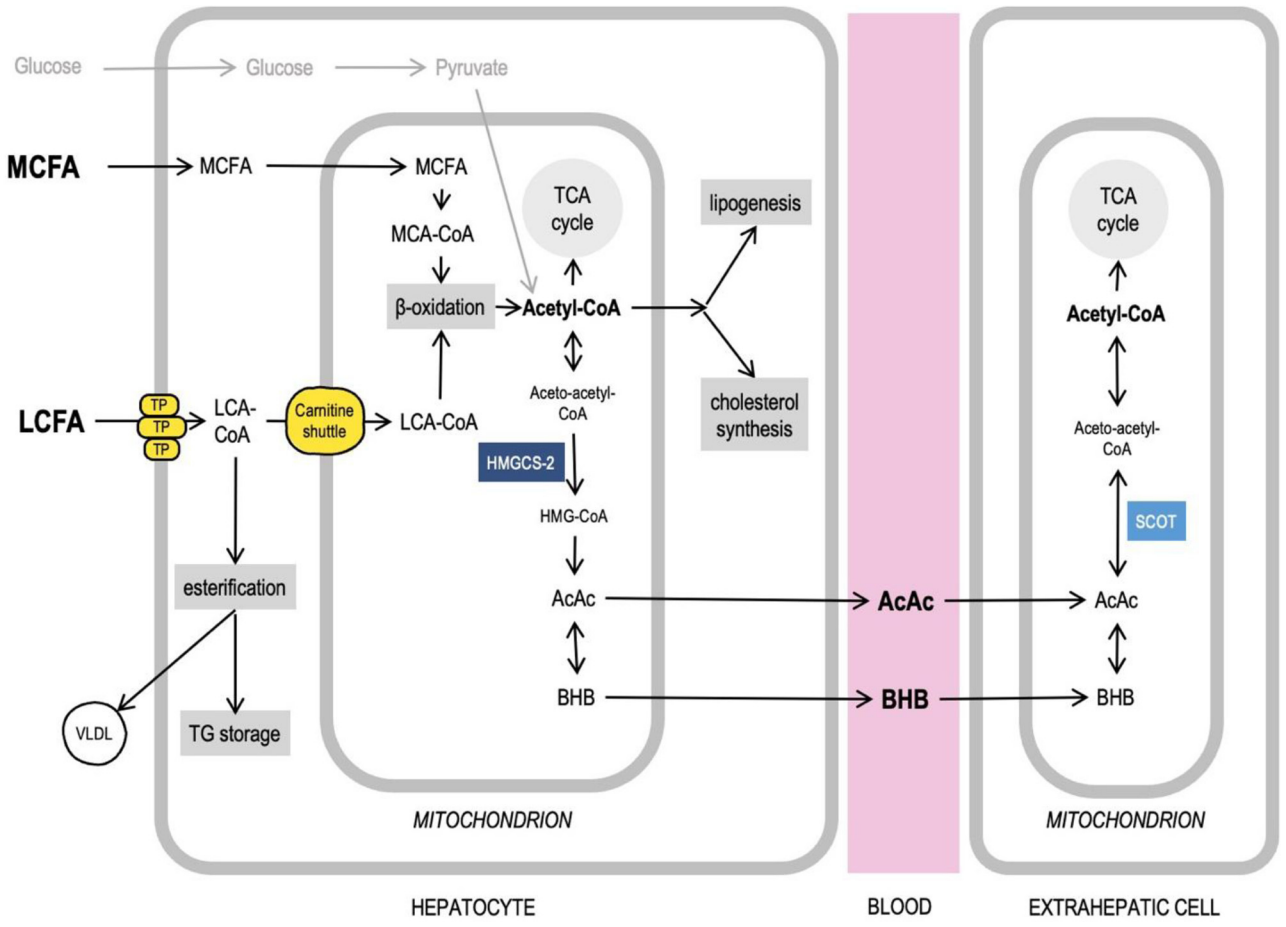
Medium-chain fatty acids, acetyl-CoA, and intersecting liver metabolic pathways. Long-chain fatty acids (LCFA) and medium-chain fatty acids (MCFA) are handled by the cells differently. MCFAs do not require transport proteins (TP) to cross membranes, get activated and undergo oxidation in mitochondria. Acetyl-CoA can feed TCA cycle, ketogenesis, lipogenesis, cholesterol synthesis. Excess LCFAs can be esterified to be stored in liver and excreted as VLDL particles. More details in text. AcAc, acetoacetate; Acetyl-CoA, acetyl-Coenzyme A; βHB, β-hydroxybutyrate; HMG-CoA, β-Hydroxy β-methylglutaryl-Coenzyme A; HMGCS-2, 3-hydroxy-3-methylglutaryl-CoA synthase 2; LCA-CoA, long-chain acyl-Coenzyme A; LCFA, long-chain fatty acids; MCA-CoA, medium-chain acyl-Coenzyme A; MCFA, medium-chain fatty acids; SCOT, Succinyl-CoA:3-ketoacid CoA transferase; TCA cycle, tricarboxylic acid cycle; TG, triglycerides; TP, transport proteins (see details in text); VLDL, very low density lipoprotein.

Medium-chain fatty acids (MCFAs) are saturated fatty acids with a chain length from 6 to 10 carbon atoms. Triglycerides that contain MCFAs are called medium-chain triglycerides. As a consumable product, MCTs are produced from coconut and palm kernel oils, both very rich in MCFAs (primarily, the caprylic (C8) and capric (C10) fatty acids) (12). Because of the difference in chain length, MCFAs and LCFAs have different physical properties (MCFA are soluble in water) and are handled by the cells differently, since many enzymes that use fatty acids as substrates are specific to certain chain lengths. After intestinal absorption, in enterocytes, LCFAs get activated (i.e., fused with CoA to form an acyl-CoA—a form in which fatty acids can enter various metabolic pathways) and esterified, trigger chylomicron formation and get transported *via* lymph, whereas MCFAs largely avoid activation and are primarily transported directly to the liver through the portal vein. In liver cells, the MCFAs, again, avoid activation in the cytosol and enter mitochondria, where they get activated to undergo β-oxidation to produce acetyl-CoA (13). The activated LCFAs can only cross the mitochondrial membranes with the help of the carnitine transport system. When glucose levels are high, insulin stimulates the acetyl-CoA carboxylase activity leading to an increased production of malonyl-CoA, which, in turn, inhibits the carnitine palmitoyltransferase I (CPT-I), one of the components of the carnitine transport system, thus significantly limiting the amount of LCFAs available for β-oxidation in a healthy, well-fed organism (14, 15). Therefore, in hepatocytes, the MCFAs are rapidly oxidized in the mitochondria, whereas the activated LCFAs are primarily directed toward triglyceride (TG) storage, phospholipid biosynthesis, and excretion in very-low-density lipoprotein (VLDL) particles (13). When enough MCFAs are available, their uncontrolled oxidation may produce amounts of acetyl-CoA, which exceed the capacity of the tricarboxylic acid (TCA) cycle. This acetyl-CoA can be redirected to various metabolic pathways, including ketogenesis in the mitochondria, as well as *de novo* lipogenesis and cholesterol synthesis in the cytosol. The ketone bodies [acetoacetate (AcAc) and β-hydroxybutyrate (βHB)], produced in the liver, can be transported with blood to other organs, including the brain, where they can be converted back to acetyl-CoA and used in the TCA cycle to produce ATP (14) (Figure 1). LCFAs are ketogenic only under conditions such as starvation and KD (when glucose is low), or diabetes. MCFAs, on the other hand, are ketogenic even in a well-fed state in the presence of carbohydrates (16, 17), which is why they can be used as a ketogenic supplement when added to a regular carbohydrate-rich diet.

A growing number of studies have demonstrated that MCT supplementation of a regular diet has a positive effect on cognition, both in healthy individuals and those suffering from mild cognitive impairment (MCI), Alzheimer's disease (AD), and other neurological disorders [for details see review (18)]. Although it is generally assumed that the procognitive effects of MCT supplementation are mediated by KB, and human studies are typically designed in line with this hypothesis, a few animal and *in vitro* studies have shown that MCFAs may also exert effects in the brain directly, as will be discussed in detail below. The mechanisms of the beneficial effects on cognition are far from being fully understood, and it is often unclear which MCT metabolites and to what extent mediate the effects of MCT supplementation.

Moreover, since MCFAs, LCFAs, and glucose participate in mutually intertwined metabolic pathways, it is important to understand how MCT supplementation of regular diet affects metabolic health in the long term. Doses used in human studies typically lie within 1 g/kg, which is generally considered safe, according to a review of MCT toxicologic properties (19). However, only several studies also monitored cardiometabolic effects alongside cognitive assessment (Table 1), and there exist reports of exceeding the 1 g/kg concentration range until the desired neuroprotective effect has been reached (47). MCTs are cheaper than KB salts and esters and are now widely commercially available. Their consumption is unregulated, and the MCT consumer behavior has not yet become a subject of systematic study.

**Table 1:** Human studies of chronic and acute administration of medium-chain triglycerides (MCT) in healthy subjects and individuals suffering from Alzheimer's disease and Mild Cognitive Impairment.

**Condition**	**Administered substance**	**Administered amount/** **concentration**	**Administration protocol**	**Measured ketone body levels**	**Measured MCFA levels**	**Cerebral and cognitive effects**	**Metabolic effects**	**Molecular effects/** **Mechanism of Action**	**References**
Elderly with mild to moderate dementia	MCT (C8) (Bulletproof Brain Octane ®)	42 g/day	Total study duration 15 months. Double blind phase: 1-month titration, 3-month therapy; Crossover arm: 1-month titration, 3-month therapy; Extension phase: 1-month titration, 6-month therapy. The first month of each phase: a week of test oil with dosing titration from 15 mL once daily to three times daily (3 x 15 mL) by week three, if clinically tolerated, or to the maximum tolerated dose. Given with meals.	**Blood:** 0.19 mM βHB (baseline) 0.22 mM βHB (end of study)	Not measured	**Improvement**: Cognigram® 1 (attention and psychomotor function), Mini-Mental State Examination (MMSE) Montreal Cognitive Assessment (MoCA) **No change:** Cognigram® 2	**No change in blood:** βHB (morning fasting level) Total cholesterol Triglyceride HDL LDL	**MMSE decliners:** were on AChEI (acetyl cholinesterase inhibitors) therapy (3 of 4) were homozygous or heterozygous for the APOE ε4 allele (4 of 4)	(20)
MCI	MCT (60% C8 + 40% C10)	30 g/day	MCT in lactose-free skim milk, twice a day, i.e. with breakfast and dinner, over a period of 6 months.	**Blood:** 0.54 mM βHB	**Blood:** 0.13 mM C8 0.16 mM C10	**Improved:** Language (Boston Naming Test) **No change:** Mini-Mental State Examination (MMSE) scores Montreal Cognitive Assessment (MoCA) scores Episodic memory tests Executive function tests Attention and processing speed tests	**Increased in blood:** βHB AcAc **No change in blood:** Total cholesterol Triglycerides Glucose Glycated hemoglobin Creatinine Thyroid stimulating hormone Vitamin B12	**Increased:** Uptake and utilization of AcAc and βHB across the whole brain **Positive linear correlation:** Plasma ketone body concentration with some of the cognitive assessment scores, including the Boston Naming Test	(21)
MCI	MCT (60% C8 + 40% C10)	30 g/day	MCT in lactose-free skim milk, twice a day, i.e. with breakfast and dinner, over a period of 6 months.	Reported previously (21)	Reported previously (21).	Cognitive scores reported previously (21). **Increased:** Functional connectivity within the dorsal attention network (DAN), Ketone uptake (11C-acetoacetate PET) specifically in DAN cortical regions, Fiber density within the DAN.	Reported previously (21).	**Improved:** Brain network energy status Axonal integrity **Positive correlation:** Functional connectivity with ketone uptake Functional connectivity with improvement in cognitive tests targeting attention	(22)
Elderly nursing home residents	MCT (75% C8 + 25% C10)	6 g/day	6 g MCTs at breakfast or dinner for 1.5 months	Not measured	Not measured	Slightly increased Mini-Mental State Examination score (P=0.06) independently of timing	Not measured	NA	(23)
Alzheimer's disease (AD)	MCT (C8 + C10), MCT (C8)	30 g/day	One month. The dose was progressively increased to reach a plateau of 30 g/day within a week and was split between 2 meals (15 g/125 mL per meal).	**Blood:** 0.46 mM βHB (MCT(C8C10)) 0.57 mM βHB (MCT(C8))	Not measured	**Increased cerebral metabolic rates (CMR):** - AcAc (C8C10 and MCT(C8): whole brain, white matter, subcortical, frontal, occipital, temporal, cingulate, gray matter), - Ketones (C8C10 and MCT(C8): whole brain). **No change:** - CMR of glucose (C8C10 and MCT(C8): whole brain, white matter, cerebellum, subcortical, frontal, occipital, temporal, parietal, cingulate, gray matter), - global or regional gray matter volume, - cortical thickness, - intra-cranial cerebrospinal fluid volume, - default mode network connectivity.	**Increased in blood:** AcAc (C8C10) βHB (C8C10), Ketones (C8C10) Insulin (C8C10 and MCT(C8)) TG (C8C10) **No change in blood:** AcAc (MCT(C8)) βHB (MCT(C8)) Ketones (MCT(C8)) Red cell count White cell count Hemoglobin Glucose Albumin ALT AST Creatinine Sodium, Potassium Chloride Cholesterol TG (MCT(C8)) HDL LDL	**Increased:** Total brain energy metabolism Ketone supply without affecting brain glucose utilization	(24)
AD	MCT (75% C8 + 25% C10)	20 g/day	MCT drink (20 g of MCT in total 39.5 g of fat, suspended in hot water) for 12-weeks along with usual diet. Blood sampling and cognitive testing: every 4 weeks. Blood sampling: -> fasting for 12 h -> blood sampling 1 -> MCT intake -> +120 min blood sampling 2.	**Blood:** 0.47 mM βHB	Not measured	**Improved:** Digit-symbol coding (Wechsler Adult Intelligence Scale-3rd Edition) Logical memory, immediate and delayed (Wechsler Memory Scale-Revised) Stroop effect (Stroop test) **No change:** Trail-making test	**Increased in blood:** βHB AcAc	NA	(25)
AD	MCT (not specified)	40 ml.	MCT: 40 ml MCT + 152 ml heavy whipping cream, Placebo: 232 ml heavy whipping cream. Two visits: -> overnight fasting -> blood sampling 1 and ApoE genotyping -> MCT intake -> +90 min blood sampling 2 -> 30-min cognitive testing -> blood sampling 3.	**Blood:** 0.52 mM βHB (+120 min; ApoE4(-)) 0.68 mM βHB (+120 min; ApoE4(+))	Not measured	**Improved:** Performance on the Alzheimer's Disease Assessment Scale-Cognitive Subscale (ADAS-cog); ApoE4(-) subjects. **No change:** Performance on the Alzheimer's Disease Assessment Scale-Cognitive Subscale (ADAS-cog); ApoE4(+) subjects.	**Increased in blood:** βHB	**βHB elevations were moderated by ApoE genotype:** ApoE4(+) - βHB continued to rise between min 90 and 120 ApoE4(-) - βHB held constant between min 90 and 120 **Positive correlation:** Ketone values with improvement in paragraph recall	(26)
AD	MCT (C8)	10 g/day	MCT-containing powder was mixed with water, milk, or juice prior to consumption for 90 days. Five study visits: Screening, Baseline, and post-baseline Days 45, 90, and 104 (± 3 days).	**Blood (+90 min):** 0.15 mM βHB (baseline post-dose) 0.36 mM βHB (Day 45 post-dose) 0.39 mM βHB (Day 90 post-dose)	Not measured	**Improved:** Performance on the Alzheimer's Disease Assessment Scale-Cognitive Subscale (ADAS-cog); ApoE4(-) subjects (Days 45 and 90). No change: Performance on the Alzheimer's Disease Assessment Scale-Cognitive Subscale (ADAS-cog); ApoE4(+) subjects.	**Increased in blood:** βHB	**Positive correlation in APOE4(-) subjects:** Total dosage of MCT with improvement in ADAS-Cog score Blood βHB level with improvement in ADAS-Cog score	(27)
AD	MCT (C8)	20 g of MCT/day	40 g/day of Axona drink (containing 20 g of C8) for 3-month	**Blood:** 0.32 mM βHB + AcAc	Not measured	*In APOE4-negative subjects with baseline MMSE score of ≥14:* **Increased:** Mini-Mental State Examination scores **No change:** Alzheimer's Disease Assessment Scale (ADAS) scores *In APOE4-negative subjects with baseline MMSE score of <14 and APOE4-positive subjects:* **No change:** Mini-Mental State Examination scores Alzheimer's Disease Assessment Scale (ADAS) scores	Not measured	NA	(28)
Mild cognitive impairment (MCI)	MCT	56 g/day	56 g of MCTs (MCT oil, Nestle™) mixed with fat-free fruit yogurt, 24 weeks	**Blood:** 0.39 mM βHB (after 4 weeks) 0.54 mM βHB (after 24 weeks)	Not measured	*In 1 (and only) APOE4-negative subject:* **Improved:** (statistical analysis was impossible due to small group size) Memory Overall cognitive assessment score In 1 (and only) APOE4-positive subject: **Improved:** (statistical analysis was impossible due to small group size) Memory	Not measured	NA	(29)
Healthy adults	MCT (60% C8 + 40% C10)	20 g/day	Overnight fasted subjects consumed two 250 mL carbohydrate-containing Peptamen® drinks (containing 10 g of MCT) 4 h apart	**Blood:** maximum ~0.28 mM at 30 min	**Maximum in Blood:** 0.15 mM at 30 min	NA	Not measured	**Increased:** Redox ratio NAD+/NADH in the brain	(30)
Type 1 diabetic patients in intensive care; insulin-induced hypoglycemia	MCT (67% C8 + 27% C10 + 6% other fatty acids)	40 g	20 g, 10 g, 10 g MCT with 25 min intervals given in 50 ml drink during stepwise hyperinsulinemic-euglycemic-hypoglycemic clamp studies	**Blood:** ~ 0.45 mM βHB (at 180 min)	Not measured	**Improved:** Hypoglycemia-induced impaired performance in tests of: - Verbal memory - Digit symbol coding - Digit span backwards - Map searching	**Increased:** plasma free fatty acids	NA	(31)
Healthy young adults	MCT (30% C8 + 70% C10)	12 or 18 g/day	12 g or 18 g MCT/day (as 6g gels 30 min prior to meals or cognitive testing; after overnight fasting when before breakfast) for 4 weeks	Not measured	Not measured	**Improved:** Trail Making A/B Digit Span Forwards/Backwards Spatial Span Backwards **No changes:** Attention Reaction time	Not measured	NA	(32)
Healthy elderly	MCT Ketogenic drink (C8 30% and C10 10% of total fatty acids)	20 g	50 g of low-carbohydrate Meiji817-B drink, single as emulsion after 12h fasting	**Blood:** 0.5 mM at 90 min	Not measured	**Improved:** Working memory Visual attention Task switching	Not measured	NA	(33)
Healthy elderly	MCT Ketogenic drink (C8 30% and C10 10% of total fatty acid)	20 g	50 g of low-carbohydrate Meiji817-B drink, single as emulsion after 12h fasting	**Blood:** ~ 0.7 mM βHB + AcAc	Not measured	**Improved:** (in subjects with reduced gray matter in dorsolateral prefrontal cortex) N-back task for attention NoGo task for inhibitory control	Not measured	**Increased:** Ketone body utilization in dorsolateral prefrontal cortex	(34)
Healthy adults	βHB	Infusion of 200 mmol/L sodium d-βHB	200 mmol/L labeled sodium D-βHB infused at a bolus rate of 16.7 ml/min for 20 min, followed by 22 μmol/kg/min for 120 min	**Blood:** 2.2 mM βHB **Brain:** 0.15-0.25 mM βHB	Not measured	NA	NA	βHB is metabolized primarily in the neuronal compartment.	(35)
Children with epilepsy. Age 18 months to 18 years.	MCT (not specified)	60% of total cal.	Given as an MCT-skimmed milk drink, in small sips throughout each meal. Total period 1–4 years.	**βHB in Blood:** *Age 2–9 years:* 2.9 mM (MCT diet < 1 month) 4.3 mM (MCT diet > 1 month) *Age 10–18 years:* 1.2 mM (MCT diet < 1 month) 1.6 mM (MCT diet > 1 month)	Not measured	A significantly greater proportion of children with mean BHB blood levels above 2 mM achieved good to excellent seizure control than did children with mean blood level <2 mM (chi-square = 5.8, P < 0.02).	**Increase:** total fatty acids in plasma (*vs pre-diet levels*), acetoacetate in plasma (in children <9 y.o.) **Decrease:** glucose in plasma; **No change:** cholesterol in plasma, blood pH, acetoacetate in plasma (in children >9 y.o.).	NA	(36)
Healthy adults	βHB	Infusion of 200 mmol/L sodium d-βHB	200 mmol/L labeled sodium D-βHB infused at a bolus rate of 80 mm/kg/min followed by an adjusted 20 mm/kg/min for the duration of the infusion study of approximately 75 min.	**Blood:** ~ 2.12 mM βHB (relatively stable from 12 to 75 min) **Occipital lobe:** ~ 0.24 mM βHB (within the first 30 min)	Not measured	Not measured	**Increase:** βHB in blood βHB in occipital lobe **No change:** Lactate level in blood Lactate in occipital lobe	NA	(37)
Children with epilepsy	MCT (81.1% C8 + 15.7% C10)	Average 45.9% of total cal. Maximum 60% of total cal.	Starvation for 1–4 days, water-restricted diet until the urine is acid, MCT slowly introduced, full diet starting day 18. MCTs are given as “Liquigen” drink (emulsion of 52% MCT + 48% water).	Not measured	**Mean in Blood (45.9% cal from MCT):** 0.31 mM C8 0.16 mM C10 **Maximum in Blood (60% cal from MCT):** 0.74 mM C8 0.55 mM C10	Not measured	Not measured	NA	(38)
Healthy adults	MCT (C8) 91% pure, MCT (C10) 91% pure MCT (55% C8 + 35% C10)	A 20 mL dose of each test oil was homogenized into 250 mL of lactose-free skim milk.	Five separate metabolic study days for each participant. 8-h metabolic study day: first 20 ml dose of the homogenized test oil taken with breakfast and a second 20 ml dose taken 4 h later without an accompanying meal.	**Maximum** **βHB in Blood:** +0.18 mM (MCT(C10)) +0.41 mM (MCT(C8C10)) +0.6 mM (MCT(C8)) **Maximum AcAc in blood:** +0.1 mM (MCT(C10)) +0.2 mM (MCT(C8C10)) +0.25 mM (MCT(C8)) **Maximum** **βHB** **+** **AcAc in blood:** +0.21 mM (MCT(C10)) +0.61 mM (MCT(C8C10)) +0.85 mM (MCT(C8))	**Maximum in blood:** + 0.1 mM C8 [MCT(C8C10)]+0.29 mM C8 [MCT(C8)] +0.1 mM C10 [MCT(C8C10)] +0.25 mM C10 [MCT(C10)]	Not measured	**Increased in blood:** βHB AcAc	NA	(39)
Traumatic brain injury patients	MCT (not specified)	23 g/1000 Kcal.	following traumatic brain injury (TBI): 1 fasting (0 Kcal; median time 37 h), 2) intermediate nutrition (7.5 Kcal/kg; median time 55 h, 3) stable nutrition 15 Kcal/kg; median time 85 h.	**βHB** **+** **AcAc in Blood:** 0.668 mM (fasting) 0.459 mM (intermediate nutrition) 0.129 mM (stable nutrition) **βHB** **+** **AcAc in Brain:** 0.0347 mM (fasting) 0.0173 mM (intermediate nutrition) 0.0131 mM (stable nutrition)	**C8 in Blood:** 0.0012 mM (fasting) 0.0182 mM (intermediate nutrition) 0.0163 mM (stable nutrition) **C10 in Blood:** 0.0079 mM (fasting) 0.0187 mM (intermediate nutrition) 0.0152 mM (stable nutrition) **C8 and C10 in Brain:** ranged between 0.001 and 0.002 mM and increased significantly during nutrition.	Not measured	*Brain (overall median values and 10-90 percentiles):* Total ketone bodies, 0.017 mM [6.1–62.6] Glutamate, 0.003 mM [0.9–24.2] Glucose, 1.1 mM [0.5–2.7] Pyruvate, 0.104 mM [65.5–166.8] Lactate, 2.9 mM [1.8–5.4] Lactate/pyruvate ratio 29 [20–46]	NA	(40)
Non-obese adults	MCT (61% C8 + 32% C10)	Formulated diet with 40% MCT or long-chain fat, 150% of estimated energy requirements	The subjects consumed the experimental diet (40% MCT or long-chain fat, 150% of estimated energy requirements) for 6 days.	Not measured	Not measured	NA	**Increased:** Triglycerides	MCT cause a significant increase in the hepatic synthesis of these fatty acids from MCFA through *de novo* synthesis and/or chain elongation and desaturation.	(41)
Healthy adults	MCT (65,8% C8 + 33,5 C10)	70 g/day	70 g of daily fat intake was replaced with MCT (or sunflower oil) for 2 weeks	Not measured	Not measured	NA	**Increased:** Total cholesterol LDL cholesterol Triglycerides Glucose	NA	(42)
Primary hypertriglyceridemic patients	MCT (72% C8 + 24% C10)	*Ad libitum* MCT:long-chain fat in different proportions	Subjects were given 500 ml bottles of oil (MCT and corn oil in different proportions) and asked to add it to their regular food. The amount of oil not consumed was measured each week.	Not measured	Not measured	NA	**No change:** HDL-cholesterol Triglycerides **Increased:** Total cholesterol	NA	(43)
Moderately overweight Chinese subjects with type 2 diabetes mellitus	MCT (not specified)	18 g/day	MCT was administered as part of daily food intake for 90 days	Not measured	Not measured	NA	**Increased:** Serum C-peptide **Decreased:** Waist circumference Body weight Insulin resistance Caloric intake **No change:** Glucose Insulin Triglycerides Total cholesterol HDL cholesterol LDL cholesterol Apolipoprotein A Apolipoprotein B	NA	(44)
Children with epilepsy	MCT (not specified)	MCT diet (60% MCT) Modified MCT diet (30% MCT)	24 h metabolic study was conducted in children 3 weeks after the diet was established.	*MCT diet:* Total plasma ketone bodies up to 2 mM *Modified MCT diet:* Total plasma ketone bodies up to 1 mM	Not measured	NA	*Both MCT diets:* **No changes:** Total cholesterol HDL cholesterol LDL cholesterol Pyruvate Lactate **Decreased:** Alanine	NA	(45)
MCI	MCT (60% C8 + 40% C10)	30 g/day	MCT in lactose-free skim milk, twice a day, i.e. with breakfast and dinner, over a period of 6 months.	**Blood**: 0.8 mM βHB (after 6 months)	**Blood:** 5.5 mg/dl C8 (after 6 months) 5.0 mg/dl C10 (after 6 months)	Reported previously (21)	**Increased:** Interleukin 8 βHB C8 C10 **No change:** Body mass index HbA1c (glycated hemoglobin) Glucose Insulin HDL, LDL, Total cholesterol Triglycerides C-reactive protein Granulocyte-macrophage colony-stimulating factor (GMCSF) Interferon gamma Interleukin 10 Interleukin 6 Interleukin 17 Interferon gamma-inducible protein 10 (IP10) Monocyte chemoattractant protein 1 (MCP1) Tumor necrosis factor-alpha (TNFα) Tumor necrosis factor-α receptor 1 (TNFR1)	NA	(46)

*AcAc, acetoacetate; AD, Alzheimer's disease; ALT, alanine aminotransferase; APOE4, apolipoprotein 4; AST, aspartate transaminase; C10, capric acid; C8, caprylic acid; HDL, high density lipoprotein; LCFA, long-chain fatty acids; LDL, low density lipoprotein; MCFA, medium-chain fatty acids; MCI, Mild cognitive impairment; MCT, medium-chain triglycerides; MMSE, Mini-Mental State Examination; NA, not applicable; βHB, β-hydroxybutyrate*.

Below we summarize the available research on the effects, MCT metabolite-specific mechanisms, and metabolic consequences of the MCT supplementation of a regular diet for the purpose of enhancing cognition, highlighting open questions and potential considerations.

## Human Studies of MCT Supplementation of Regular Diet

Over the past few years, several human studies explored whether MCT supplementation-induced ketogenesis was sufficient to achieve measurable effects on cognition and brain energy metabolism. In this section, we discuss the studies of MCT supplementation in patients with MCI and AD, and healthy individuals, summarizing the dose, administration protocol, the type of MCFAs used in MCT formulation, major outcomes, and the relationship between the observed effects and the KB plasma levels. The details of the listed human studies can be found in Table 1. For a meta-analysis of MCT supplementation studies in MCI and AD, the reader may be referred to Avgerinos et al. (18).

### Studies in Subjects With Mild Cognitive Impairment

In a randomized, double-blind, placebo-controlled crossover study, 6 months followed by another 6 months of open-label extension of MCT (C8) supplementation with up to 42 g a day (25.2 g on average), given with meals, improved some cognitive assessment scores in elderly subjects with mild to moderate dementia (20). In another study in MCI subjects, 6 weeks of MCT supplementation (30 g / day, C8+C10) improved verbal fluency scores compared to placebo (21). Although within the group receiving MCT, the scores in several other cognitive tests have improved compared to the values before the intervention, there was no difference compared to placebo. MCT consumption increased the cerebral metabolism of KB across both cortical and subcortical regions, and the degree of cognitive improvement in some cognitive tests correlated with the brain KB uptake. An analysis of resting-state functional connectivity across eight brain networks in the MCI subjects in this clinical trial was published in a separate paper (22), demonstrating that after MCT supplementation, the connectivity in one of the eight networks, the dorsal attention network (DAN), was 59% higher compared to the placebo group, which was also associated with better scores in some cognitive tests (22). Improved DAN connectivity was associated with increased ketone body uptake and plasma levels post-administration. In a 1.5-month intervention trial, the addition of 6 g of MCT (C8+C10) to either breakfast or dinner was reported to have improved scores in the Mini Mental State Examination test in elderly nursing home residents, although the significance level of this finding was at P=0.06 (23).

### Studies in Alzheimer's Disease Patients

A PET study in Alzheimer's Disease (AD) patients demonstrated that 1 month of daily consumption of 30 g of MCT (C8+C10 or C8) led to mild ketonemia and increased total brain energy metabolism due to an increase in ketone body utilization (24). In another open-label placebo-controlled study, although ingestion of a drink containing 20 g of MCT (C8+C10) had no effect on cognition in AD patients, 12 weeks of daily consumption of this drink together with a regular diet led to significant improvements in logical memory tests (25).

Several studies of MCT supplementation in AD patients showed that the intervention efficacy depended on APOE4 status. APOE4 is an allelic variant of Apolipoprotein E, a lipid-binding protein that helps transport fatty acids in the brain between astrocytes and neurons. APOE4 variant disrupts normal fatty acid transport and metabolism in the brain and is a strong genetic factor for late-onset AD (48). Although i*n vitro* βHB has been shown to rescue energy defects associated with the APOE4 isoform (49), several human studies found that APOE4-positive individuals benefited less from ketogenic interventions. For example, a single 38 g oral dose of MCT (95% C8, 5% C10) given after an overnight fast in a low-carb-high-fat drink improved cognitive performance (memory, but not attention) assessed at the peak of the ketonemia in APOE4-negative AD patients, and the degree of improvement correlated with the βHB levels (26). However, although APOE4-positive AD patients in the same study developed even higher levels of βHB and its levels remained high longer than in APOE4-negative patients, however, no cognition-enhancing effect was observed. In a randomized double-blind placebo-controlled study, 3 months of supplementation with 20 g MCT (C8) daily improved cognitive measures in APOE4-negative but not APOE4-positive AD patients (27). In another open-label study in a Japanese population, similarly, 3 months of supplementation with 20 g MCT (C8) improved cognitive assessment scores in APOE4-negative (but not in APOE4-positive) AD patients with higher baseline scores but failed to improve cognition in individuals with more advanced progression of the disease (28). Additionally, in a double-blind randomized placebo-controlled parallel study in individuals diagnosed with MCI with a total number of 4 participants who completed the trial (out of whom 2 received MCT), 24 weeks of MCT supplementation (56 g/day, C8+C10) improved memory and overall AD cognitive assessment scores in one APOE4-negative subject and memory in one APOE4-positive subject (29). The two control subjects in this study demonstrated no memory or overall score improvements. As no statistical analysis was possible due to the small sample size, this report on memory improvement in an APOE4-positive individual should be taken with caution.

### Studies in Cognitively Healthy Subjects

The literature on ketogenic supplementation has been primarily concerned with its clinical applications to alleviate some of the symptoms of neurodegenerative and other cognitive diseases. Therefore, the data in healthy subjects is limited. However, it has been demonstrated that a single ingestion of 10 g of MCT (C8+C10) increased the NAD+/NADH redox potential in the brain of healthy volunteers by 18% (30). When intensively treated type 1 diabetic patients with normal cognition received 40 g of MCT (C8+C10) during a controlled period of hypoglycemia, this intervention attenuated the hypoglycemia-induced impairment in cognitive performance (31). Administration of either 12 or 18 g of MCT (C8+C10) for 3 weeks improved cognitive performance in healthy young adults without significant dose-dependent differences (32). Several studies have also demonstrated benefits for elderly individuals not diagnosed with any cognitive impairment. In a double-blind placebo-controlled study, a single ingestion of 20 g of MCT (C8+C10) improved working memory and attention in elderly individuals with normal cognition (33). An fMRI study in healthy elderly individuals found that a single 20 g dose of MCT (C8+C10) improved performance in some cognitive tests, and some of these improvements correlated with an increased rate of KB utilization in the dorsolateral prefrontal cortex (34).

Thus, it appears that the addition of 12–56 g of MCT to a regular diet can lead to significant cognitive improvements, often correlated with the measured elevation of KB. Although the utilization of KB has been reported to double across all brain regions (21), it is unclear why MCT supplementation improves performance in some cognitive tests but not the others. A lower dose of 12 g was sufficient to improve cognition in one study, and a single 10 g dose has significantly increased the NAD+/NADH ratio in the brain. However, very few studies used doses lower than 20 g. Cognitive assessment tests are typically conducted at the peak of the elevated plasma KB levels, based on the assumption that an immediate availability of KB must be required to improve task performance. However, while certain procognitive effects can be observed after a single administration, other effects require continuous chronic administration, suggesting the involvement of different mechanisms. Very few studies measured MCFA concentrations, and none estimated their correlation with cognitive testing results. Most studies focused on aged individuals and those suffering from cognitive decline. It appears that once the cognitive decline has progressed to a certain stage, MCT supplementation becomes less effective. Therefore, MCT supplementation may be recommended as a preventative measure to help sustain cognition before the decline. Healthy younger subjects may benefit from MCT supplementation to the same extent as healthy elderly subjects, if not more. More studies are needed to clarify the effects of MCT supplementation on cognition in healthy young adults.

## The Mechanisms of the Procognitive Effects of MCT Supplementation

Most human studies of the procognitive effects of MCT supplementation have been designed to determine whether MCT supplementation can be clinically used to alleviate cognitive deficits or to improve cognitive function (Table 1), and the effects were typically assumed to be mediated by ketone bodies. Several clinical studies in dogs followed this line of investigation and demonstrated that MCT supplementation can be used to reduce the frequency of seizures and improve cognitive performance in dogs (50–54). A number of rodent and *in vitro* studies revealed that not all the effects of MCT supplementation could be linked to liver ketogenesis and investigated the mechanisms of these effects. In the following section, we review the known mechanisms of MCT supplementation and MCFA action focusing on metabolite-specific effects.

### Region and Phenotype-Specificity

Two studies demonstrated that the effect of MCT feeding on brain energy metabolism in specific brain regions was different depending on the presence or absence of stressful conditions.

Brownlow et al. compared the effects of a MCT ketogenic diet (MCT-KD, 27% w/w MCT (C8) with the protein/fat/carbohydrate caloric ratio of 22,5/77/0,5) with a ketogenic supplementation (KS) protocol (MCT (C8) and βHB salts mixture added to regular chow, 10% w/w) (55). This study was meant to assess whether nutritional ketosis could provide cognitive benefits in healthy adult rats with and without accompanying stress (induced by hypoxia). In the MCT-KD-fed animals, βHB stayed chronically elevated at about 1.3 mM, while the KS feeding increased the βHB levels for 4 h post-administration with a subsequent return to baseline. Notably, neither ketogenic treatment affected the βHB concentrations measured in the hippocampus. Conversely, chronic stress reduced the hippocampal βHB concentrations in all animal groups, although this reduction was less pronounced in the MCT-KD group. The finding that the hippocampal βHB level was more affected by stress than ketogenic intervention indicates that the ketone body metabolism likely plays an important role in the hippocampus. Both MCT-KD and, although to a lesser extent, KS significantly upregulated the levels of β-hydroxybutyrate dehydrogenase-1 (BDH1) and acetyl-CoA transferase (ACAT1) in the hippocampus. BDH1 participates in both biosynthesis and utilization of the ketone bodies, while ACAT1 is a mitochondrial enzyme which catalyzes a reversible conversion between acetoacetyl-CoA and 2 molecules of acetyl-CoA—a reaction step in both acetoacetate utilization and the final step of β-oxidation (56, 57). This pattern of enzyme level change in the brain together with the observation that even the MCT-KD had no effect on the brain βHB concentration could potentially be interpreted as a sign of an increase in MCFA β-oxidation in the brain. The hippocampal level of GLUT-1 (endothelial and astrocytic glucose transporter) was lower in the MCT-KD and KS groups compared to control. GLUT-3 (neuronal glucose transporter) hippocampal protein levels were not affected by either ketogenic treatment. Chronic stress reduced GLUT-1 and elevated GLUT-3 levels in the hippocampus, while both ketogenic treatments completely abolished this effect. Finally, both the MCT-KD and KS protocols attenuated the stress-induced decrease of BDNF (brain-derived neurotrophic factor) in the hippocampus (p ≤ 0.05). A similar effect was recently demonstrated in mice, where MCT-KD attenuated the decrease in cortical levels of BDNF induced by a high-fat-high-cholesterol non-ketogenic diet (58).

Hollis and colleagues investigated the effects of 2 weeks of MCT supplementation on anxiety in rats (59). They divided adult male rats into groups based on the anxiety-like behavior (high, normal, and low anxiety). MCT (C8+C10) was mixed into the chow (5% of caloric intake). Under this supplementation protocol, the blood βHB concentration significantly increased from 0,10–0,15 mM to 0,15–0,25 mM. The brain concentration of βHB was not measured. MCT supplementation reduced anxiety in highly anxious animals, but not in animals with normal anxiety levels. The treatment stimulated dominant behavior in animals with varying baseline anxiety level but did not affect depressive-like behavior. MCT supplementation reduced mitochondrial respiration and the Complex I protein levels in the prefrontal cortex (PFC) of the high-anxiety animals to the levels observed in low-anxiety animals. In the same high-anxiety animals, MCT supplementation elevated the intracellular levels of GLUT-1, GLT-1 [also known as EAAT-2, glutamate transporter found in astrocytes and neurons, responsible for glutamate reuptake (60)], and Na+/K+ ATPase subunits (which support the function of GLT-1). No changes were documented in the PFC of low-anxiety animals. Similarly, no changes were found in the Nucleus accumbens of animals of either anxiety level.

The results of these two studies indicate that the MCT supplementation may affect the energy metabolism and neurotransmission in the brain, however the effects may be region- and phenotype-specific. For example, the level of GLUT-1, a transporter required for glucose transport across the blood-brain-barrier, was modulated by the MCT supplementation differently depending on the investigated brain structure and other factors, such as stress or anxiety. More studies are needed to clarify the significance of brain βHB levels under various conditions irrespective of the feeding regime. More studies should also focus on comparing the effects of MCT supplementation among different brain regions.

### Differential Effects of C8 and C10

Historically, most MCT studies have been carried out using MCTs containing a mixture of caprylic (C8) and capric (C10) fatty acids. However, the past decade of research has revealed significant differences in the effects of C8 and C10 both *in vitro* and *in vivo*.

**Figure 2:**
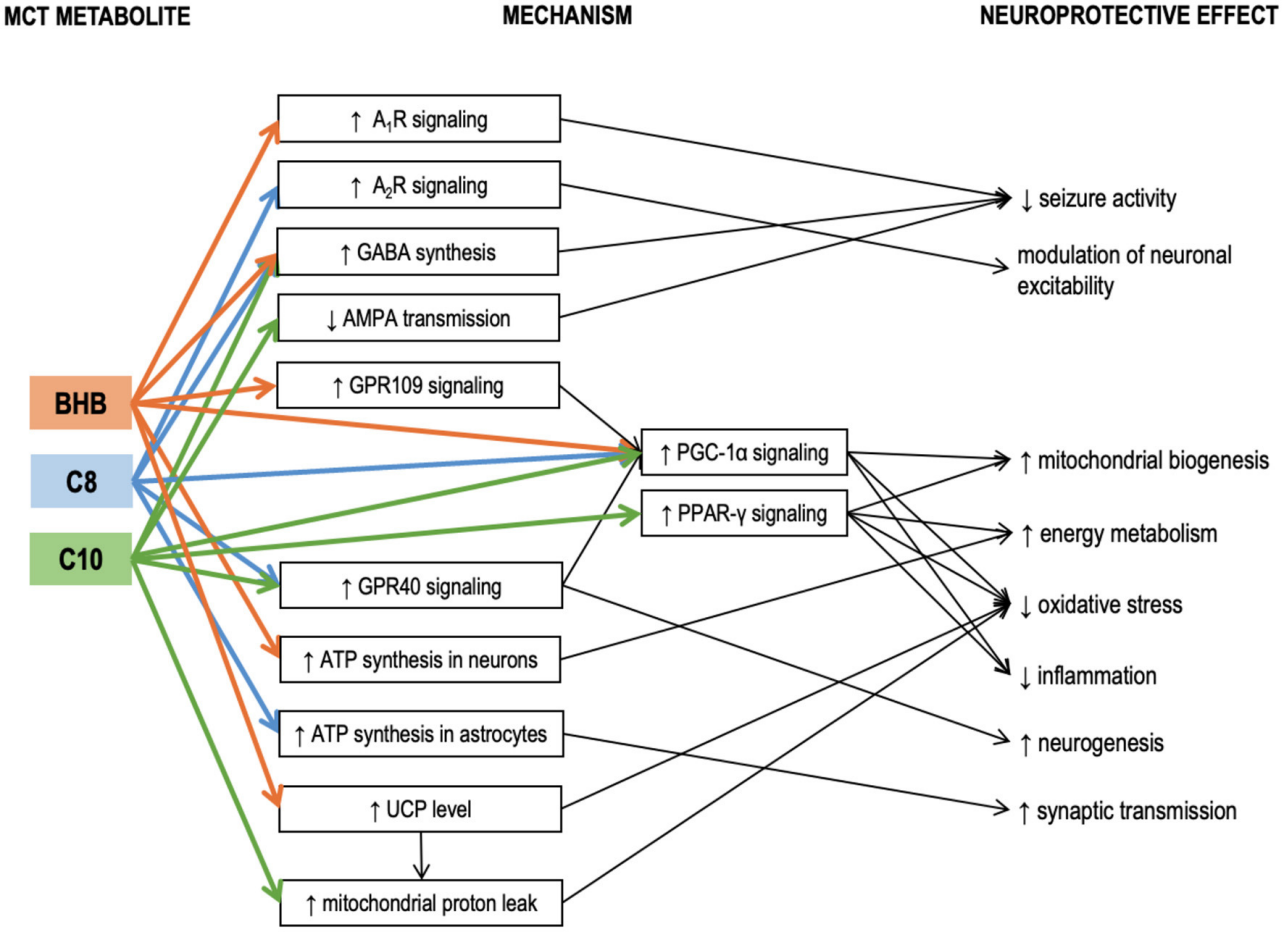
Known mechanisms of the neuroprotective effects of medium-chain triglyceride (MCT) metabolites – caprylic (C8) and capric (C10) medium-chain fatty acids and β-hydroxybutyrate. Details in text. A1R, A2R, adenosine receptors A1 and A2; AMPA, α-amino-3-hydroxy-5-methyl-4-isoxazole propionic acid (AMPA receptor is a subtype of glutamate receptor); βHB, β-hydroxybutyrate; C10, capric acid; C8, caprylic acid; GABA, γ-aminobutyric acid; GPR109, G-protein-coupled receptor 109; GPR40, G-protein-coupled receptor 40; MCT, medium-chain triglycerides; PGC-1α, Peroxisome proliferator-activated receptor γ coactivator 1α; PPAR-γ, Peroxisome proliferator-activated receptor γ; UCP, uncoupling proteins.

Hughes et al. incubated neuronal cell line SH-SY5Y cells with either C8, or C10, or βHB for 6 days (61). Only C10 increased the activity of citrate synthase (the enzyme of the first reaction of the TCA cycle), mitochondrial Complex I activity, and catalase activity. Electron microphotographs of the C10-treated cells revealed an increase in number and decrease in the size of mitochondria. Co-incubation with an inhibitor of PPAR-γ (peroxisome proliferator-activated receptor-gamma) abolished the C10 effect on the citrate synthase activity. Therefore, C10 (but not C8 and not the ketone bodies) enhanced energy metabolism and triggered mitochondrial proliferation in this neuronal cell line through interaction with PPAR-γ. This finding is in line with the reports demonstrating that C10 binds with PPAR-γ well, while C8 – very poorly (62). The ability of C10 to activate citrate synthase in a PPAR-γ-mediated manner has also been confirmed in human fibroblasts (63). Additionally, ketogenic diet has been shown to reduce seizure activity in mice *via* a mechanism involving PPAR-γ (64). PPAR-γ is a ligand-inducible transcription factor expressed in many organs, including the brain, where it is predominantly found in microglia (65). Microglia and astrocytes tend to increase PPAR-γ production during neuroinflammation (66). Saturated fatty acids longer than C8 can act as ligands activating PPAR-γ to trigger transcription of its downstream genes (67). PPAR-γ mediates transcription of antioxidant enzymes such as superoxide dismutase and catalase and inhibits various proinflammatory and inflammatory pathways, including the NF-kB (nuclear factor kappa-light-chain-enhancer of activated B cells) pathway (65, 68, 69). PPAR-γ agonists demonstrated anti-inflammatory properties in various models of neuroinflammatory and neurodegenerative diseases, both *in vitro* and *in vivo* (65, 66, 70–73). Therefore, PPAR-γ activation may be one of the neuroprotective mechanisms of the KD and MCT supplementation, which is not directly linked to βHB, but may be acted upon with C10 (Figure 2).

Tan et al. compared the effects of chronic administration of MCT (C10) and MCT (C8) in 2 murine models of epilepsy (6Hz test and injection of flurothyl, a GABA_A_ receptor blocker) (74). A diet with 35% calories consumed in the form of MCT (C10) had a significant anticonvulsant effect, while a similar diet with MCT (C8) had no effect. While neither blood nor hippocampal βHB levels were affected by either of the MCT diets, the blood levels of the corresponding MCFAs were elevated. MCT (C10) diet also significantly increased the brain C10 concentration, but no such effect was found in the MCT (C8)-fed animals, suggesting the anticonvulsant effect of the MCT (10) diet could be mediated by the C10 effects in the brain (74). MCT (10) diet increased the plasma antioxidant capacity and upregulated the mRNA expression of several antioxidant enzymes in the hippocampus, including the *Hmox1* (heme oxygenase 1). Although *Hmox1* transcription is known to be activated by Nrf2 (nuclear factor erythroid 2-related factor 2) (75), which, in turn, may be activated by βHB (76), other antioxidant enzyme genes downstream to Nrf2 were not upregulated, thus further suggesting that βHB was not responsible for the observed effects of the MCT (10) diet. In the same study, when C8 or C10 were added to cultured astrocytes, both MCFAs stimulated basal respiration in mitochondria indicating that astrocytes could oxidize both these MCFAs. However, only C10 increased proton leak. Proton leak, also referred to as uncoupling, is a net movement of protons across the inner mitochondrial membrane not associated with ATP production. Although protein leak decreases ATP production per one mitochondrion, consistent uncoupling has been shown to trigger mitochondrial proliferation and thus a net increase in ATP per cell in the hippocampus, protecting mice against seizures (77). Proton leak also decreases ROS production (78). Therefore, its elevation protects brain cells against oxidative stress (79). A recent study in mouse brain slices likewise confirmed that C8 oxidation was linked to ATP production, while C10 oxidation was linked to increased proton leak (80). Uncoupling proteins (UCPs) are implicated in the mechanism of protein leak (81). Notably, although in the study by Tan et al., C10 stimulated proton leak in cultured astrocytes, no increase in proton leak was registered in the mitochondria isolated from the hippocampus of the MCT-fed animals. Additionally, the MCT (10) diet did not affect the hippocampal expressions of uncoupling protein genes *Ucp2, Ucp3, Ucp4*, and *Ucp5*. It is essential to highlight that UCPs are not always conducting protons but are tightly regulated. UCPs are activated by fatty acids (82). C12 and C14 have the strongest stimulating effect on proton transport. C10 has a weaker effect, while C8 and shorter fatty acids are unable to activate UPCs (83). This may explain why several studies have confirmed that C10 but not C8 was able to stimulate protein leak in mitochondrial preparations. It has been demonstrated that ketogenic diet can increase both the levels and the activity of uncoupling proteins in the brain (84). On the other hand, βHB ester consumption has been reported to increase UCP4 and UCP5 protein levels in the brain, accompanied by an increase in brain levels of malonyl-CoA (85). Although the proton leak was not measured in this study, the accumulation of malonyl-CoA should increase fatty acid synthesis and thus activate UCPs. βHB could potentially increase the levels of uncoupling proteins *via* its effect on peroxisome proliferator-activated receptor-gamma (PPAR-γ) coactivator (PGC)-1α [a transcription coactivator, which controls mitochondrial biogenesis and UCP expression (86, 87)]. Therefore, the lack of *ucp* upregulation in the study by Tan's group is consistent with the lack of βHB elevation. It is unclear why C10 stimulated the proton leak *in vitro* but not *in vivo*. A potential explanation could be that the brain concentration of C10 was not high enough. Further studies are needed to determine whether C10-induced protein leak could be promoted by MCT feeding. To summarize, it appears that βHB can increase the UCP protein expression in the brain, while C10 may potentially activate UCPs to increase the proton leak (Figure 2). These two mechanisms may act together in KD and when taking ketogenic doses of C10-containing MCTs.

MCT consumption has also been linked to PGC-1α in another study, where a single and, to an even greater extent, a repeated intragastric administration of MCT (C8, 0.15 g/kg) reduced the toxic effects of MPTP (1-methyl-4-phenol-1,2,5,6-tetrahydropyridine) in a murine model of Parkinson's disease (88). MPTP is known for its ability to selectively damage dopaminergic neurons. Intraperitoneal injection of MPTP significantly reduced the levels of dopamine and its metabolites in the striatum. MCT administration 1.5 h before the MPTP injection allowed to reduce its toxic effect. Single MCT administration increased striatal mRNA level of PGC-1α. PGC-1α is required for induction of many reactive oxygen species (ROS)-detoxifying enzymes and has been shown to grant neuroprotection against MPTP and kainic acid in other studies (89). A potential mechanism of PGC-1α induction is the activation of GPR109 (G-protein coupled receptor 109, also known as hydroxycarboxylic acid receptor 2, HCA2) by its ligand βHB (90–92). Signaling through GPR109 is involved in some neuroprotective effects of KD and βHB (91). However C8 itself may also increase PGC-1α expression through its interaction with another G protein-coupled receptor GPR40 (also known as free fatty acid receptor 1, FFAR-1), known to be activated by MCFAs [although C8 shows lower affinity compared to C10 (93)]. GPR40 signaling has been associated with neurogenesis (94), potentially participating in signaling cascades involving the phosphorylated-cyclic adenosine monophosphate response element-binding protein (pCREB) and BDNF (95). *In vivo* administration of MCFA to mice excited a subpopulation of pro-opiomelanocortin (POMC) neurons in the hypothalamus, and this effect was mediated through GPR40 (96). Therefore, βHB and MCFAs could both act to upregulate PGC-1α, triggering antioxidant response and improving cellular energetics through mitochondrial biogenesis (Figure 2).

When various medium-chain fatty acids were compared in their potency to suppress pentylenetetrazol (PTZ) induced epileptiform activity in rat hippocampal slices, C10 was found to be more effective than valproic acid (VPA), a commonly used anti-epileptic drug; C9 (pelargonic fatty acid) was slightly less effective than C10; C8 had no effect (97). *In vitro*, the anti-seizure effect of C10 could be achieved at 0.1 mM concentration. In the same study, intraperitoneal injection (0.4 g/kg) of C9 (C10 and C8 were not studied) 10 min after the onset of status epilepticus established in awake rats was more effective in suppressing seizures than VPA (97). As valproic acid is a well-known teratogen due to its inhibitory effect on HDAC (histone deacetylases) (98), the authors of the study monitored dose-dependent effects of MCFAs on HDAC activity (97). C8 and C10 had no effect in physiological concentrations (2 mM), although at higher concentrations C10 had a stronger HDAC-inhibitory activity than C8. Therefore, in this model, C10 and C8 were very different in terms of their anticonvulsant activity. In a later study by the same group, C10 but not βHB prevented seizure activity in rat hippocampal slices in two different epilepsy models. The study also revealed that C10 can act as an antagonist of AMPA receptors (99) to suppress seizure activity (Figure 2). The effect of MCT supplementation on AMPA transmission has been confirmed *in vivo* in a study by our group, where 4 weeks of daily MCT (C8+C10, 2 ml/kg) administration during 6 h of fasting improved spatial and working memory in healthy rats, accompanied by increased expression of the GluN2a- and GluN2b NMDA receptor subunits and the GluA1- and GluA2- AMPA receptor subunits in the medial PFC (100, 101). If C10 inhibits glutamatergic signaling through AMPA receptors, upregulation of the receptor level may be required to sustain glutamatergic transmission necessary for learning.

Wlaz et al. published two similar reports on the effects of single intragastric administrations of C8 (102) and C10 (103) on seizure threshold in several murine epilepsy models assessed in a range of standard tests. C8 (30 mg/kg) was effective against myoclonic and clonic but not tonic seizures induced by PTZ injection, while C10 (30 mg/kg) had no effects in the same tests. Both C8 and C10 (10-30 mg/kg) were effective in the 6-Hz seizure test. Neither C8 nor C10 (30 mg/kg) were effective in the maximal electroshock test. Co-administration of C8 and C10 at therapeutic concentrations had an additive effect, which may be indicative of at least partially different mechanisms of action (102, 103).

One of the mechanisms of the C8 anticonvulsant properties seems to be mediated through adenosine receptors. It is well established that elevated extracellular adenosine reduces seizure activity in several seizure models, and the anticonvulsant action is mediated through A_1_R adenosine receptors (104, 105). One of the main factors controlling the amount of extracellular adenosine is the adenosine kinase (ADK) (105). KD reduces ADK activity which results in increased extracellular adenosine and suppressed seizure activity (106). Although it has been demonstrated in hippocampal slices that the reduction in excitability can be achieved through ATP/pannexin-1/adenosine/A_1_R/K_ATP_ pathway simply by reducing the glucose concentration in the buffer without any addition of βHB (105), a series of studies in Wistar Albino Glaxo/Rijswijk (WAG/Rij) rats, a genetic model of absence epilepsy, conducted by D'Agostino, Kovacs and colleagues revealed that βHB itself seems to be able to elicit anticonvulsant effects through a mechanisms involving A_1_R. Using a mixture of ketone salts and medium-chain triglycerides (KSMCT) as the ketogenic supplement (2.5 g/kg/day), they demonstrated that administration of A_1_R antagonist abolished the anti-seizure effect (i.e., decrease in number of spike-wave discharges – SWD) established by 7 days of KSMCT treatment (107) and dose-dependently decreased or abolished the anxiolytic effects of KSMCT treatment (108). In both studies, KSMCT treatment significantly elevated the blood level of βHB. In another study, they showed that the same KSMCT supplementation protocol alleviated isoflurane-induced anesthesia (immobility), and this effect was mediated through A_1_R but not A_2_R receptors (109). This observation was further confirmed in yet another study by the same group, where KEKS food [standard chow with mixed in ketone esters and ketone salts (110)] supplementation decreased the lipopolysaccharide-induced elevation of SWD in the same rat model, while A_1_R, but not A_2A_R antagonism abolished the anti-seizure effect of KEKS supplementation (111). In contrast to this series of studies, in a mouse seizure model, 30 mmol/kg (5 g/kg) C8 (administered as free fatty acid) increased the seizure threshold in the 6 Hz psychomotor seizure threshold test, and this effect was blocked by both A_1_R and A_2A_R antagonists (112), suggesting that C8 may affect adenosine signaling through a mechanism not involving βHB. The involvement of A_2_R was further confirmed in another study, where the effects exerted by C8 on POMC neurons were abolished with A_2a_R and A_2b_R antagonists (96). Therefore, both KD and ketogenic supplementation of regular diet seem to have neuroprotective effects mediated by A_1_R and possibly also A_2_R receptors (Figure 2). It is possible that increased ATP synthesis due to influx of KB or MCFAs in the brain results in elevated extracellular adenosine concentrations, which act upon AR (105, 113), although the exact mechanism is unknown.

Wang and Mitchell compared the effects of 8 weeks of supplementation (5% of caloric intake) with either MCT (C8) or MCT (C10) in aged rats and found differences on both behavioral and biochemical tests (114). The plasma concentrations of MCFAs reached 6,4 μM (C8) and 18,2 μM (C10). Similar to the Tan's group results in mice, the brain level of βHB did not differ among the two MCFA and the LCFA control groups. In behavioral tests, both MCT (C8) and MCT (C10) enhanced social recognition, however only MCT (C10) improved new object recognition. Locomotor activity was decreased in animals in the MCT (C8)-fed group but not in the MCT (C10)-fed group. Protein expression of Ube-3a (ubiquitin protein ligase 3A) was elevated 2-fold in the PFC of the MCT (C10)-fed animals and 4-fold in the PFC of the MCT (C8)-fed animals compared to control. Ube-3a plays an important role in protein degradation and it has been shown that its shortage in the hippocampus of aged rats is associated with memory impairment (115). The mRNA levels of many immediate early genes (IEGs), including *Arc, Erg1, Erg2, Junb, Plk3, Nr4a1*, and *Fosb* were reduced in the PFC of the MCT-fed animals. The effects of C8 and C10 containing MCTs were very similar, except for *Junb* whose level was significantly lower in the MCT (C8)-fed group. As this is one of the first reports on the effects of MCT on neuroplasticity, it is quite difficult to interpret these results. In studies concerning the molecular mechanisms of memory and learning, the expression of IEGs, such as *Arc, Erg1, Erg2, Junb, Fosb*, is usually measured immediately after learning or an acute stress event. In such experiments, elevated expression of an IEG is interpreted as its involvement in neuroplasticity. Although the role of elevation of decrease of the basal level of IEG mRNA is less clear, some studies have linked increased basal expression of *Arc* in the hippocampus of aged rats with spatial memory deficits (115). In other words, elevated basal level of IEG expression may signal a reduced sensitivity of the IEG induction in response to incoming stimuli. The observed property of MCTs to decrease basal level of IEGs in PFC requires further research. Wang and Mitchel also measured the levels of the hippocampal growth factors VEGF (vascular endothelial growth factor), GDNF (glial cell-derived neurotrophic factor), and IGF-1 (insulin-like growth factor 1), but found no effect of MCT supplementation (114).

To summarize, a few studies investigated the differential roles of C8 and C10 on brain physiology. C10, but not C8, directly modulates AMPA receptors activity, activates PPAR- γ and facilitates, at least *in vitro*, the mitochondrial proton leak. The two MCFAs differ in their effects on seizure activity, although the results are controversial for different models and feeding regimes. The mechanism of anticonvulsant effects of C8 may be related to signaling through A_2a_R and A_2b_R adenosine receptors. The effects of MCT supplementation on neurotransmission and neuroplasticity-related genes cannot be easily explained by global effects such as increased ATP production or reduced oxidative stress and require more detailed studies. Additionally, more studies should implement experimental designs allowing to distinguish between the effects of βHB and the two MCFAs. The details of the studies related to differential effects of C8 and C10 can be found in **Table 3**.

### MCFA Oxidation and Ketogenesis in the Brain

It is widely accepted that adult mammals' brain uses almost no LCFA (C14-C18) in energy metabolism (121, 122). However, it seems that MCFAs, if administered/present in the diet, may be used by the brain to a far greater extent. The brain is physically protected with the blood-brain barrier (tight junctions between endothelial cells). Therefore, fatty acids must cross cellular membranes to get inside the brain. In the brain, similar to other tissues, LCFA transport across membranes requires various proteins, such as fatty acid transport protein (FATP), fatty acid translocase (FAT)/CD36, plasma membrane-bound fatty acid binding protein (FABPpm), and cytosolic fatty acid-binding protein (FABPc) (123). Only a small amount of LCFAs injected into rat's carotid artery is taken by the brain (124). On the other hand, MCFAs are soluble in water, do not require transport proteins to cross membranes, and predominantly avoid activation in the cytoplasm by acyl-CoA synthases. 94% of C8 and 88% of C10 injected into the carotid artery are taken by the brain (125). When injected directly into CSF, C8 rapidly crosses the blood-brain barrier into the peripheral blood, whereas LCFA transport is limited (96).

Primary cultures of astrocytes from new-born rats oxidize fatty acids (C8, C14, C16) (116, 117). It has been noted that since β-oxidation enzymes are more active in developing brain, the higher-than-expected rates of β-oxidation in some studies could have been an artifact due to the use of cells obtained from a developing brain or stem cells (126). However, β-oxidation is required for neural progenitor cells proliferation in an adult brain's hippocampus (126). Human astrocytes obtained from adult epileptic patients undergoing neurosurgery readily oxidized C16, and fatty acid oxidation was required for neuroprotection following an ischemic stroke in adult mice's hippocampus (118). A recent study in adult mouse brain slices revealed that C8 and C10 were preferentially metabolized and not stored in astrocytes (80). The metabolism of both MCFAs supported glutamine synthesis, which in turn supported neuronal GABA synthesis. In astrocyte preparations, astrocytes oxidized C8 more actively than βHB, while neurons oxidized βHB 3-fold more actively than astrocytes but were unable to oxidize C8 (117). Modeling in a human magnetic resonance study estimated that βHB oxidized is neuronal compartment is approximately 1.85 times more actively than in the astrocytic compartment (35). Therefore, the emerging view is that oxidation of βHB and MCFAs may be largely compartmentalized to neurons and astrocytes, respectively. Consistent with this view, in rat hippocampal slices, βHB but not C8 could support synaptic transmission under hypoglycemic conditions, although C8 added together with βHB was able to improve the rate of recovery of synaptic function after the glucose concentration was restored (31). Therefore, MCFAs and βHB may act in the brain synergistically to support synaptic function. However, contrary to this generalization, a few studies reported that neuron-like cells also were able to oxidize MCFAs. In a human neuronal cell line (originally derived from neuroblastoma), the rate of C8 oxidation was 5-fold greater compared to C10, albeit much lower compared to glucose (119). C8 was also oxidized in a cell line derived from mouse embryonic hypothalamic neuronal primary culture (96). Further studies are required to clarify whether neurons are able to oxidize MCFAs *in vivo* or *ex vivo*.

When rats were injected with sodium salt of C8, as much as 20% of the brain's acetyl-CoA was determined to be a product of the injected C8's oxidation (127), although the contribution of the liver ketogenesis to the brain's acetyl-CoA production was not directly accounted for, and the interpretation of the results of this study has been questioned (128). When fatty acids were administered to mice by oral gavage, unlike LCFA, C8 was actively oxidized in the hypothalamus and affected the firing rates of POMC neurons, exhibiting an excitatory effect in some populations and inhibitory in others (96). Therefore, MCFA oxidation has been experimentally confirmed *in vivo*. Additionally, medium-chain acyl-CoA dehydrogenase (MCAD, an enzyme participating in β-oxidation of both MCFAs and LCFAs when they are shortened to medium-chain length) deficiency, one of the most prevalent disorders of fatty acid oxidation, may lead to accumulation of C8 and C10 in the brain (129). MCAD has been found in both developing and adult rat brain in much greater quantity than LCFA-specific acyl-CoA-dehydrogenases, which may be indicative of the brain's ability to oxidize larger quantities of MCFAs (130). The existence of this disease suggests that a healthy brain may be oxidizing significant amounts of MCFAs. Therefore, although fatty acid oxidation may account for only a small amount of ATP produced in the brain, it may play an important not-yet-understood role in various brain regions.

In addition to being able to carry out β-oxidation, astrocytes can also produce ketone bodies (116, 120, 131–134). Ketogenic diet increases transcription of *hmgcs-2* (hydroxy-3-methylglutaryl-CoA synthase 2, the key enzyme of ketogenesis) in the brain (135). Both in the liver and primary astrocyte culture, ketogenesis was more active with C8 as a substrate compared to C16 (120). Although, quantitatively, astrocytes cannot compete with the liver in the quantity of produced ketone bodies, brain ketogenesis may play region-specific regulatory role. For example, astrocytic ketogenesis in the ventromedial hypothalamus is involved in control of food intake in response to a high-fat diet regimen (133).

Therefore, existing data indicate that MCFAs may be oxidized directly in the brain and act as a substrate for ketogenesis in the brain, much more so than LCFAs. These effects of MCFAs in the brain, as well as their relative importance, require further research. Known neuroprotective mechanisms linked to MCT metabolites βHB, C8, and C10 are summarized in Figure 2.

### Therapeutic Concentrations of βHB and MCFAs

It is reasonable to assume that if a chemical molecule exerts certain effects, the occurrence of these effects should correlate with the concentration of this molecule. Although the neuroprotective effects of ketogenic diet and MCT supplementation have been predominantly considered as being mediated by βHB, some studies found no correlation between the βHB concentration and the extent of the neuroprotection offered by KD or MCT supplementation, which brought more attention to investigating the direct effects of MCFAs.

In one study, a plasma βHB concentration of 4 mM correlated with the anticonvulsant effect of KD in children (136). In another study, the anticonvulsant effect was found to be “good” or “excellent” when βHB concentration was above 2 mM (36). On the other hand, when C10 was chronically administered to mice in two different seizure models, the anticonvulsant effect did not correlate with βHB concentration (74). MCT added to regular food (9% of daily caloric intake) reduced seizure frequency without significant βHB elevation in dogs (53). In clinical studies of MCT supplementation, the therapeutic effect on cognition was achieved after oral administration of 38 g (26) and 20 g (27) of MCT resulting in the elevation of plasma βHB levels to, correspondingly, 0.43–0.68 mM and 0.30–0.40 mM, which is an order of magnitude less than the therapeutic concentrations reported in the KD studies.

The brain concentrations of βHB are rarely measured in the studies of neuroprotective effects of MCT (Tables 1, 2), however a few studies have looked specifically at the relationship between the plasma and brain concentrations. Intravenous administration of βHB to healthy adult volunteers elevated plasma βHB level from 0.2 mM when fasted in the morning to 2.1 mM following the administration. Using magnetic resonance spectroscopy, βHB concentration in the occipital lobe was estimated as 0.24 mM, about 10-fold lower than in the blood (37). Fasting for 2 and 3 days brought the βHB levels, correspondingly, to 1.7 mM and 3.2 mM in plasma and 0.6 mM and 1.0 mM in the brain, about 3-fold lower than in the blood (37). Although there is about 3-fold difference between the values given by the two studies, starvation is known to increase brain uptake of the KB (143), and ketogenic diet is known to increase the monocarboxylate transporter (required for KB transport) expression in the brain (144), which may explain why the measured values were higher in starved subjects.

**Table 2:** Animal studies of chronic and acute administration of medium-chain triglycerides (MCTs) and their metabolites caprylic (C8) and capric (C10) medium-chain fatty acids and β-hydroxybutyrate.

**Object**	**Model/** **Condition**	**Administered** **substance**	**Administered** **amount/** **concentration**	**Administration** **protocol**	**Measured** **ketone** **body** **levels**	**Measured** **MCFA levels**	**Cerebral** **and Cognitive** **effects**	**Metabolic** **effects**	**Molecular effects** **/Mechanism** **of Action**	**References**
Beagle dog	Aged dogs (8–11 years)	AC-1203 MCT (95% C8 + 5% C10).	2 g/kg Added to standard feed	2 months	Not measured	Not measured	Not measured	Not measured	**Increased in parietal cortex:** Total phospholipid DHA (Docosahexaenoic acid) DPA (Docosapentaenoic acid) Total n-3 polyunsaturated fatty acid	(50)
Dog	Aged Beagle dogs	MCT (97% C8 + 3% C10)	MCT supplement, 5.5% w/w mixed into the food made by Nestle· Purina PetCare	8 months	**βHB in Blood:** 0.11 mM	Not measured	**Improved:** Visuospatial function Learning Reversal learning Attention More difficult tasks showed more significant effects.	**No change:** Standard dog blood panel parameters	NA	(51)
Dog	Aged dogs with canine cognitive dysfunction syndrome (analogous to dementia in people)	MCT (FA content unspecified)	Standard diet containing 6.5% or 9% MCT	3 months	**βHB in Blood:** No difference from control (measured fasting level)	Not measured	**Decreased:** Signs of cognitive dysfunction syndrome	**Increased in Blood:** DHA (Docosahexaenoic acid) EPA (Eicosapentaenoic acid) total omega-3 polyunsaturated fatty acids omega-3/omega-6 ratio **No change in Blood:** Cholesterol Glucose Total triglycerides	NA	(52)
Dog	Dogs diagnosed with idiopathic epilepsy	MCT (60–65% C8, 30–50% C10)	9% of cal	3 months supplementation 7 days washout 3 months supplementation	**βHB in Blood:** 0.070 mM (preprandial) 0.059 mM (postprandial)	Not measured	**Decreased:** Seizure frequency Seizure day frequency	**Decreased:** Blood alkaline phosphatase activity **No change:** Body weight Blood glucose Pancreas lipase activity	NA	(53)
Dog	Dogs diagnosed with idiopathic epilepsy	MCT (60–65% C8, 30–50% C10)	9% of cal	3 months supplementation 7 days washout 3 months supplementation	Not reported	Not measured	**Improved:** Spatial-working memory Problem-solving Owner-reported trainability	Not measured	**Positive correlation:** Problem-solving test results with the postprandial blood βHB	(54)
Rat	2 m.o. Sprague Dawley rats Chronic hypoxia-induced stress	MCT (C8)-rich ketogenic diet (**KD-MCT**) 20g KetoCaNa in 100 ml MCT (C8) (**KS**)	*KD-MCT:* 27% w/w MCT(C8) added to standard feed (F/C/P: 77.0/0.5/22.5) *KS:* 10% (w/v) added to standard feed	**Chronic:** 3 weeks transition into diets + 3 weeks ad libitum **Acute:** Single intragastric 10 g/kg KS	**βHB in Blood:** *Acute:* ~ 2.6 mM (peak at 1h) *Chronic:* ~ 1.3 mM (*KD-MCT*) ~ 0.5 mM (*KS*) **βHB in Hippocampus:** *With no stress:* ~ 0.9 mM (*KD-MCT*) ~ 0.9 mM (*KS*) ~ 0.9 mM (*ctrl*) *Under stress:* ~ 0.6 mM (*KD-MCT*) ~ 0.5 mM (*KS*) ~ 0.45 mM (*ctrl*)	Not measured	*KD-MCT:* **Increased (all regardless of the stress):** Novel object exploration Spatial learning Spatial memory (MWM Probe trial) **No change:** Passive avoidance *KS:* **Increased:** Spatial learning (on day 4 impaired by stress) **No change:** Novel object exploration Passive avoidance Spatial memory (MWM Probe trial)	*KD-MCT:* **Increased:** Peripheral fat pad βHB **Decreased:** Body weight Glucose Insulin **No change:** Epydimal fat pad ACTH, CORT (basal and restrain stress-induced) *KS:* **Increased:** Peripheral fat pad Epydimal fat pad **No change:** Body weight Glucose βHB, Insulin ACTH, CORT (basal and restrain stress-induced)	*KD-MCT:* **Increased:** BDH1 (*vs ctrl & KS*, stress-induced increase) Hippocampal ACAT1 **Decreased:** Hippocampal GLUT1 (*vs ctrl & KS*, no stress induced reduction) **No change:** Hippocampal βHB (but partially attenuated stress-induced reduction) Hippocampal BDNF Hippocampal GLUT3 (basal) *KS:* **Increased:** Hippocampal ACAT1 β-hydroxybutyrate dehydrogenase-1 (BDH1) **Decreased:** Hippocampal GLUT1 (no stress induced reduction) **No change:** Hippocampal βHB (stress induced reduction) Hippocampal BDNF Hippocampal GLUT3 (basal)	(55)
Mouse	78 w.o. C57BL/6 males	MCT (FA content unspecified) ketogenic diet	MCT diet: ketogenic diet with caloric proportion of 84% fat and 2% carbohydrate	High-fat-high-cholesterol (not ketogenic, 40% fat) diet: 16 days, after that for 8 weeks: high-fat-high-cholesterol diet or high-fat-high-cholesterol diet + metformin or MCT-rich diet	**βHB in Blood:** ~5.5 (units unspecified; MCT group) ~2.6 (units unspecified; control group)	Not measured	**Improved:** Spatial learning and memory	**No change in Blood:** Glucose TG Total cholesterol Insulin AST activity (Aspartate aminotransferase) ALT activity (Alanine aminotransferase)	**Both MCT-enriched diet and adding Metformin to the high-fat-high-cholesterol diet:** Reversed the high-fat diet-induced increase (to control levels) in protein levels of: cortical and hippocampal NF-κB, cortical TNF-α, cortical and hippocampal glial fibrillary acidic protein (GFAP), cortical and hippocampal glial phosphate tau phosphate tau amyloid protein precursor (APP). MCT feeding reversed the high-fat diet-induced decrease in cortical BDNF protein levels.	(58)
Rat	2–3 m.o. Wistar males Divided into Low and High Anxiety subgroups	MCT (40% C8 + 60% C10)	5% MCT	Added to standard chow 8–15 days ad libitum	**Blood:** ~ 0.144–0.288 mM βHB	Not measured	**Decreased:** Anxiety (Dark-Light Box); **No change:** Depressive-like behavior (FST).	**Increased:** βHB in blood **No change:** Body weight Food intake	**Mitochondrial respiration:** Decreased in mPFC No change in Nucleus accumbens **Increased in mPFC:** GLUT1 (*only in High Anxiety group*) GLT1 (EAAT2) (*only in High Anxiety group*) Na/K ATPase (*only in High Anxiety group*) Hexokinase mt/cyt (*only in High Anxiety group*) **Decreased in mPFC:** phospho-GSK-3α/GSK-3α (*only in High Anxiety group*) Hexokinase (*only in Low Anxiety group*) **No change in mPFC:** GLUT1 (*only in Low Anxiety group*) GLT1 (EAAT2) (*only in Low Anxiety group*) Na/K ATPase (*only in Low Anxiety group*) Hexokinase (*only in High Anxiety group*) phospho-GSK-3α/GSK-3α (*only in Low Anxiety group*) Hexokinase (*only in Low Anxiety group*)	(59)
Mouse	7–8 w.o. CD1 males Seizure model	MCT (C8) MCT (C10)	35% of cal	Added to standard chow 10 days ad libitum	**Blood:** 0.5–1 mM βHB **Brain:** 175 nmol/g βHB	**Blood:** 0.033 mM C8 (*MCT(C8)*) 0.076 mM C10 (*MCT(C10)*) **Brain:** 2.88 nmol/g C8 (*MCT(C8)*) 1.17 nmol/g C10 (*MCT(C10)*)	**Increased:** Seizure threshold (6-Hz) [*MCT(C10)*] Tolerance to fluorothyl [*MCT(C10)*]	**Increased:** Plasma reducing capacity (anti-oxidant effect) [*MCT(C10)*] **No change:** Body weight Blood βHB Brain βHB Blood glucose	**Increased:** Hippocampal mRNA levels of heme oxygenase 1 and FoxO [*MCT(C10)*]. **No change:** mRNA levels of FoxO3, FoxO6, Sirt1 mRNA levels of Ucp2, Ucp3, Ucp4 and Ucp5 Catalase and superoxide dismutase activities in the hypothalamus Proton leak in the mitochondria isolated from the hippocampus	(74)
Rat	Adult Wistar males	3HB-BDE (R-3-hydroxybutyrate-R-1,3-butanediol monoester)	30% of cal	Added to standard chow 14 days ad libitum	**Blood:** 2.8 mM βHB	Not measured	Not measured	**Increased:** Blood ketone bodies **Decreased:** Food intake Blood glucose Blood insulin Blood leptin **No change in blood:** total fatty acids stearic acid pH	**Increased in the whole brain:** malonyl-CoA UCP4 and UCP5 protein [NAD+]/[NADPH] ratio **Decreased in the whole brain:** 3-phosphoglycerate l-lactate l-glutamate GABA **No change in the whole brain:** TCA cycle intermediates ATP hydrolysis	(85)
Mouse	12 m.o. C57BL/10Tar males MPTP model of Parkinson's Disease	C8	0.15 g/kg	**Intragastric gavage:** single (1.5 h before MPTP) repeated (3 days, 1.5 h before MPTP and 2 consecutive days)	Not measured	Not measured	Not measured	NA	**Increased in striatum:** Dopamine (*acute and repeated C8 vs. MPTP*) DOPAC (3,4-Dihydroxyphenylacetic acid) (*acute and repeated C8 vs. MPTP*) HVA (Homovanillic acid) (*acute and repeated C8 vs MPTP*) PGC-1α mRNA (Peroxisome proliferator-activated receptor-γ coactivator) (*acute C8 vs. ctrl, 1.5 h post-gavage*) PEPCKc mRNA (Phosphoenolpyruvate carboxykinase, Cytosolic) (*acute C8 vs. ctrl, 1.5 h post-gavage*) PEPCKm mRNA (Phosphoenolpyruvate carboxykinase, Mitochondrial) (*acute C8 vs. ctrl, 1.5 h post-gavage*)	(88)
Mouse	6 w.o. C57Bl/6J males	MCT (C8)	10% of cal	Single intragastric gavage	Not measured	Not measured	Not measured	**Decreased:** Food intake	**Increased in PVH (paraventricular nucleus of hypothalamus):** α-MSH (alpha melanocyte-stimulating hormone) c-fos (marker of neuronal activity)	(96)
Mouse	6 w.o. C57Bl/6J males	C8	NA	Labeled C8 was administered: - ICV: 2 μL of 1 mCi/mL - *via* carotid artery: 5 min (40 μL/min) - oral gavage: 100 μM	Not measured	Not measured	Not measured	**Increased in hypothalamus (30 min after ICV administration, 15 min after administration** ***via*** **the carotid artery, 60 min after oral gavage):** FA oxidation FA oxidation to storage ratio	NA	(96)
Rat	Wistar Han male.	High-fat diet	High-fat diet (42% fat)	Ad libitum 20 weeks	Not measured	**Blood:** ~ 0.003 mM C8 **CSF:** ~ 0.0024 mM C8	Not measured	**No change:** Total FA levels. Plasma FA levels are 2.5-fold higher than in CSF. MCFA/Total FA proportion: 1% in plasma, 4% in CSF. Among the MCFAs, 78% was C8 in both plasma and CSF.	NA	(96)
Rat	7 m.o. Wistar males	MCT (60% C8 + 40% C10)	2 g/kg/day	Intragastric daily gavage + fasting 6 h/day 4 weeks	Not measured	Not measured	**Increased:** Spontaneous alternations in Y-maze Time in target quadrant in MWM probe trial	Not measured	**Increased in mPFC:** GluN2a mRNA (NMDA receptor subunit 2A) GluN2b mRNA (NMDA receptor subunit 2B) GluA1 mRNA (AMPA receptor subunit 1) GluA2 mRNA (AMPA receptor subunit 2)	(100)
Rat	7 m.o. Wistar males	MCT (60% C8 + 40% C10)	2 g/kg/day	Intragastric daily gavage + fasting 6 h/day 4 weeks	Not measured	Not measured	**Increased:** Spontaneous alternations in Y-maze Time in target quadrant in MWM probe trial	Not measured	NA	(101)
Mouse	Adult naïve Albino Swiss males 25–30 g Seizure tests	C8	5 mmol/kg 10 mmol/kg 20 mmol/kg 30 mmol/kg	Single dosage C8 was suspended in a 0.5% aqueous solution of methyl cellulose and administered by gastric gavage (10 ml/kg) 30 min before the test.	**Blood (20 mmol/kg C8):** 0.9 mM βHB	Dose-dependent increase. **Blood:** 0.18 to 0.51 mM C8 (*5-30 mmol/kg C8*) **Brain:** 0.11 to 0.25 mM C8 (*5-30 mmol/kg C8*)	**Increased (dose-dependently):** seizure threshold (PTZ: myoclonic twitch and clonus) seizure threshold (6-Hz) **No change:** seizure threshold (PTZ: tonus) seizure threshold (MEST) C8 increased anticonvulsant potency of valproic acid (VPA) in the 6-Hz and MES seizure tests.	**Decreased in blood:** Glucose (*20 mmol/kg C8*) **No change in blood:** pH (*20 mmol/kg C8*)	NA	(102)
Mouse	Adult naïve Albino Swiss males 25–30 g Seizure tests	C10	10 mmol/kg 30 mmol/kg 50 mmol/kg	Single dosage C10 was suspended in a 0.5% aqueous solution of methyl cellulose and administered by gastric gavage (10 ml/kg) 30 min before the test.	**Blood (30 mmol/kg C10):** 1.66 mM βHB	**Blood:** 0.41 mM C10 (*30 mmol/kg*) **Brain:** 0.24 mM C10 (*30 mmol/kg*)	**Increased:** Seizure threshold (6-Hz) (dose-dependently, *10 and 30 mmol/kg*) Seizure threshold (MEST) (dose-independently, *50 mmol/kg*) **No change:** Seizure threshold (PTZ)	**Increased in blood:** βHB (30 mmol/kg) **Decreased in blood:** pH (*30 mmol/kg*) Glucose (*30 mmol/kg*)	NA	(103)
Rat	10 m.o. Wistar Albino Glaxo/Rijswijk males	MCT (60% C8 + 40% C10) + Ketone Salt (Na/K-βHB) (1:1; **KSMCT**)	2.5 g/kg/day	Intragastric gavage 7 days	**βHB in Blood:** 1.8 mM (day 1) 1.9 mM (day 7)	Not measured	**Decreased:** Spike-wave discharges (SWD) (between days 3 and 7 of gavage)	**Increased:** Blood βHB	**Inhibition of Adenosine receptor A1:** abolished the anti-seizure effect of KSMCT. The SWD number and βHB levels returned to the baseline levels on the first day without ketone supplementation.	(107)
Rat	8 m.o. Wistar Albino Glaxo/Rijswijk males	MCT (60% C8 + 40% C10) + Ketone Salt (Na/K-βHB) (1:1; **KSMCT**)	2.5 g/kg/day	Intragastric gavage 7 days	**βHB in Blood:** 1.23 mM (day 1) 1.23 mM (day 7)	Not measured	**Decreased:** Anxiety (Elevated plus maze)	**Increased:** Blood βHB **Decreased:** Blood glucose **No change:** Body weight	**Inhibition of Adenosine receptor A1:** did not change blood βHB levels modified (abolished) the anti-anxiety effect of KSMCT	(108)
Rat	6 m.o. WAG/Rij males Isoflurane-induced anesthesia (immobility) Inhibition of Adenosine A1 receptor	MCT (60% C8 + 40% C10) + Ketone Salt (Na/K-βHB) (1:1; **KSMCT**) MCT (60% C8 + 40% C10) + Ketone Ester (1,3-butanediol - acetoacetate diester) (1:1; **KEMCT**)	2.5 g/kg/day	Intragastric gavage 7 days After 7 days, isoflurane (3%) was administered for 5 min	**Blood (day 7):** 1.33 mM βHB (*KSMCT*) 2.14 mM βHB (*KEMCT*)	Not measured	**Increased:** Latency to isoflurane-induced immobility	**No change:** Body weight Blood glucose	**Inhibition of Adenosine receptor A1:** abolished MCT-evoked delay in the onset of isoflurane-induced anesthesia	(109)
Rat	10 m.o. Wistar Albino Glaxo/Rijswijk males	**KEKS food**: 10% w/w KE/R,S-1,3-butanediol-acetoacetate diester + 10% w/w KS/Na+ and Ca2+-ketone salt mixed with standard chow 1% saccharine	20% KEKS food	10 days ad libitum	**βHB in Blood:** 1.25 mM (day 1) 1.35 mM (day 10)	Not measured	**Decreased:** Spike-wave discharges (SWD) (between days 7 and 10 of treatment) Total time of SWD LPS-evoked increase in SWD number **No change:** Discharge frequency within SWD Average SWD duration Total time of sleep-waking stages	**Increased:** Blood βHB **No change:** Blood glucose Body weight	The SWD number and βHB levels returned to the baseline levels on the second day without ketone supplementation.	(110)
Rat	10 m.o. Wistar Albino Glaxo/Rijswijk males	**KEKS food**: 10% w/w KE/R,S-1,3-butanediol-acetoacetate diester + 10% w/w KS/Na+ and Ca2+-ketone salt mixed with standard chow 1% saccharine	20% KEKS food	9 days ad libitum	**βHB in Blood:** 1.25 mM (day 1) 1.40 mM (day 9)	Not measured	**Decreased:** Spike-wave discharges (SWD) (between days 3 and 9 of treatment) LPS-evoked increase in SWD number	**Increased:** Blood βHB **No change:** Blood glucose Body weight	**Inhibition of Adenosine receptor A1:** abolished the alleviating effect of KEKS food on LPS-generated increases in the SWD number. **Inhibition of Adenosine receptor A2A:** did not significantly modify the alleviating effect of KEKS food on LPS-generated increases in the SWD number.	(111)
Mouse	Adult naïve Albino Swiss males 25–30 g Inhibition of Adenosine A1 and A2a receptors	C8	20 mmol/kg 30 mmol/kg	Single dosage C8 was suspended in a 0.5% aqueous solution of methyl cellulose and administered by gastric gavage (10 ml/kg) 30 min before the test.	**Blood (*****30 mmol/kg C8*****):** ~ 2.5 mM βHB (non-fasted mice) ~ 3.8 mM βHB (fasted mice)	Not measured	**Increased:** 6 Hz seizure threshold (*30 mmol/kg*) **No change:** 6 Hz seizure threshold (*20 mmol/kg*)	**Decreased in blood:** Glucose (non-fasted mice) **No change in blood:** Glucose (fasted mice) pH (non-fasted and fasted mice)	Inhibition of Adenosine receptors A1 or A2a abolished the anticonvulsant effect of C8 (*30 mmol/kg*). Combined administration of an adenosine transporter inhibitor dipyridamole and 20 mmol/kg caprylic acid raised the threshold for the 6 Hz-induced seizures. K_ATP_ channel blockage by glibenclamide did not abolish the anticonvulsant effect of C8 (*30 mmol/kg*). Glucose (2 g/kg) abolished the anticonvulsant effect of C8 (*30 mmol/kg*) in non-fasted but not in fasted mice.	(112)
Rat	21 m.o. Wistar males Aged animals	MCT (C8) MCT (C10)	5% MCT	Added to standard chow 8 weeks ad libitum	**Blood:** ~ 0.3 mM βHB (*MCT(C10)*) ~ 0.25 mM βHB (*MCT(C8)*)	**Blood:** 0.0064 mM C8 (*MCT(C8)*) 0.0182 mM C10 (*MCT(C10)*) 0.002 mM C8 (*MCT(C10)*) 0.000 mM C10 (*MCT(C8)*)	**Increased:** Social recognition Novel object recognition (*MCT(C10)*) **Decreased:** Locomotor activity (*MCT(C8)*)	**Decreased:** Body weight **Increased in blood:** C8 (*MCT(C8) vs ctrl and MCT(C10)*) C10 (*MCT(C10) vs ctrl and MCT(C8)*) **No change in blood:** C8 (*MCT(C10) vs ctrl*) C10 (*MCT(C8) vs ctrl*)	**Increased:** pIRS-1/IRS-1 (Insulin Receptor Substrate 1) in forebrain (*MCT(C8)*) pAkt/Akt (Serine/Threonine Kinase) in forebrain (*MCT(C10)*) SYP protein (Synaptophysin) in forebrain (*MCT(C8)*) UBE3A protein (Ubiquitin-protein ligase E3A) in forebrain (*MCT(C8) and MCT(C10)*) mRNA of plasticity-related early genes in mPFC (*grin1, gba2*) **Decreased:** pS6K/S6K (Ribosomal protein S6 kinase) in brain (*MCT(C8) and MCT(C10)*) mRNA of plasticity-related early genes in mPFC (*arc, erg1, erg2, junb, plk3, nr4a1, fosb*) **No change:** GDNF protein (Glial cell line-derived neurotrophic factor) in forebrain IGF-1 protein (Insulin-like growth factor 1) in forebrain VEGF protein (Vascular endothelial growth factor) in forebrain PSD-95 protein (Postsynaptic density protein 95) in forebrain mRNA of plasticity-related early genes in mPFC (*jnk1, srf*)	(114)
Rat	Adult Wistar females and males Intracarotid infusion of C8 and C10	C8 C10	0.043 mM C8 0.022 mM C10	Intracarotid infusion Decapitation after 15 s	Not measured	NA	Not measured	Not measured	**Brain uptake:** 94% (C8) 88% (C10)	(125)
Rat	Adult Sprague Dawley males Intracarotid infusion of C8	C8	220 mM	**Intracarotid infusion (2.67 ml/h):** Unlabeled C8 (30 min) and Labeled C8 ([2,4,6,8-13C4]octanoate) (105 min)	**Blood:** 0.1312 mM βHB + AcAc (0 min) 0.3574 mM βHB + AcAc (105 min)	**Blood at 105 min:** undetectable (unlabeled C8) 0.25 mM (labeled C8)	NA	**Increased in blood:** ketone bodies (*105 min vs 0 min*) **No change in blood:** Glucose (*105 min vs 0 min*)	Oxidation of 13C-octanoate in the brain accounted for ~20% of total brain oxidative energy production.	(127)
Rat	Adult Wistar males	MCT (70% C8 + 30% C10)	8.55% C8 + 3.16% C10	Added to standard chow 4 weeks (given daily at the beginning of the dark phase)	Not measured	Not measured	Not measured	**Increased:** Apparent fat digestibility (*vs ctrl & LCT*) Liver weight (*vs ctrl*) Liver TG content (*vs ctrl & LCT*) Muscle TG content (*vs ctrl*) **Decreased:** Feed intake (*vs ctrl & LCT*) Blood TG (*vs LCT*) Blood free fatty acids (*vs ctrl*) Blood glucose (*vs LCT*) **No change:** Body weight (*vs ctrl & LCT*) Blood TG (*vs ctrl*) Blood glucose (*vs ctrl*) Blood insulin (*vs ctrl & LCT*) Liver weight (*vs LCT*) Muscle TG content (*vs LCT*) Wet weight of epididymal adipose tissue (*vs ctrl & LCT*) Wet weight of retroperitoneal adipose tissue (*vs ctrl & LCT*)	**No change:** Skeletal muscle peroxisomal oxidation Liver peroxisomal oxidation Skeletal muscle CPT-1 and CPT-2 activity Liver CPT-1 and CPT-2 activity	(137)
Rat	Sprague Dawley males. Non-alcoholic steatohepatitis (NASH).	MCT (FA content unspecified)	70% MCT	Added to chow 21 days ad libitum	Not measured	Not measured	Not measured	**Increased:** Blood adiponectin **Decreased:** Liver TG accumulation Blood leptin **No change:** Blood TG Blood insulin	**Decreased:** Hepatic TNF mRNA and protein **No change:** Hepatic CYP2E1 protein (Cytochrome P450 2E1)	(138)
Rat	Sprague Dawley males	MCT (FA content unspecified)	25% Wt MCT (45% cal MCT)	Semiliquid MCT diet given *via* a gastrostomy tube twice a day for 6 weeks (first 3 weeks–gradually increasing dosage 17–30 ml/day)	Not measured	Not measured	Not measured	**Increased:** Resting oxygen consumption Norepinephrine-stimulated oxygen consumption **Decreased:** Body weight Size of adipocytes Dissectible fat **No change:** Adipocyte density Liver fat Blood glucose	NA	(139)
Rat	Sprague Dawley males Streptozotocin-induced diabetes	MCT (FA content unspecified)	5%, 15%, 25% MCT	Added to standard chow Gradual increase: 5% for 14 days 15% for 12 days 25% for 12 days	**βHB in Blood:** *Non-diabetics:* ~ 0.11 mM (*5% MCT*) ~ 0.22 mM (*15% MCT*) ~ 0.48 mM (*25% MCT*) *Diabetics:* ~ 1 mM (*5% MCT*) ~ 8 mM (*15% MCT*) ~ 12 mM (*25% MCT*)	Not measured	Not measured	**Increased in Blood:** Ketones (*in non-diabetics vs ctrl non-diabetics*) **Decreased in Blood:** TG (*in non-diabetics vs ctrl non-diabetics*) glycerol (*in diabetics vs ctrl diabetics*)	NA	(140)
Rat	3 w.o. Lewis males weighing ~50 g	MCT (FA content unspecified)	23.4% C8, 16.9% C10	Added to standard chow 6 weeks ad libitum	Not measured	Not detected	Not measured	**Decreased in Blood:** TG Chylomicron VLDL **No change in Blood:** Cholesterol	NA	(141)
Rat	Sprague Dawley males	MCT (65% C8 + 35% C10) BMS (Na/K-βHB mineral salt) + MCT	5–10 g/kg/day	Daily intragastric gavage 28 days	**Maximum** **βHB in Blood:** ~3.8 mM (*5 g/kg MCT, 1 h after gavage*) ~5 mM (*10 g/kg MCT, 1-8 h after gavage*) ~2 mM (*5 g/kg BMS+MCT, 4 h after gavage*) ~3 mM (*10 g/kg BMS+MCT, ~4-8 h after gavage*)	Not measured	Not measured	**Increased:** Blood βHB (acutely, *MCT-containing supplements vs ctrl*) Relative liver weight **Decreased:** Body weight (*3-4 weeks, all supplements vs ctrl*) Blood glucose (acutely, *MCT-containing supplements vs ctrl*) Blood HDL (after 28 days) Relative spleen weight **No change:** Blood total cholesterol Blood TG Blood LDL Relative weight of brain Relative weight of lungs Relative weight of kidneys Relative weight of heart	NA	(142)

*AcAc, Acetoacetate; ACAT1, Acetyl-CoA acetyltransferase 1; ACTH, Adrenocorticotropic Hormone; BDH1, β-hydroxybutyrate dehydrogenase-1; BDNF, Brain derived neurotrophic factor; βHB, β-hydroxybutyrate; C10, capric acid; C8, caprylic acid; CORT, Corticosterone; CPT, carnitine palmitoyltransferase; EAAT2, Excitatory amino acid transporter 2; FA, Fatty acid; GLT1, Glutamate transporter 1; GLUT, Glucose transporter; GSK-3α, Glycogen synthase kinase-3 alpha; HDL, High density lipoprotein; KS, Ketone salt; LPS, Lipopolysaccharide; MCFA, medium-chain fatty acids; MPTP, 1-methyl-4-phenyl-1,2,3,6-tetrahydropyridine; NA, not applicable; SWD, Spike-wave discharges; TG, Triglycerides; TNF, Tumor necrosis factor; UCP, Uncoupling protein; VLDL, Very low density lipoprotein*.

In relation to MCFAs, although a large portion of orally administered MCFAs is metabolized in the liver, the plasma concentrations of MCFAs also become elevated. When children were fed a diet with MCT (C8+C10) providing 46% of caloric intake, the plasma concentrations of C8 and C10 reached 0.31 and 0.16 mM (38). A single oral dose of MCT (C10) or MCT (C8) given to mice resulted in C10 concentrations up to 0.41 mM in plasma and 0.24 mM in the brain (103) and C8 concentrations of 0.51 mM in plasma and 0.25 mM in the brain, the difference between the plasma and the brain being about 2-fold (102). In rats, cerebrospinal fluid (CSF) concentration of FA was measured to be 2.5-fold lower than in blood. However, MCFAs accounted for 1% of all FA in the blood, but 4% in the CSF (96). Therefore, based on the limited available data, it appears that the brain concentration of MCFAs may be roughly estimated to be as high as about half of their plasma concentration. Most *in vitro* studies of MCFA effects used concentrations (Table 3) comparable to the brain concentrations either measured or estimated based on their plasma concentrations in animal (Table 2) and human (Table 1) studies.

**Table 3:** *In vitro* and *ex vivo* studies of caprylic (C8) and capric (C10) medium-chain fatty acids.

**Object**	**Model/** **Condition**	**Administered** **substance**	**Administered** **amount/** **concentration**	**Administration** **protocol**	**Molecular** **effects/** **mechanism** **of action**	**References**
Brain slices	Rat hippocampal slices	βHB C8	8 mM βHB 8 mM C8	C8 or βHB were added for 30 min to the brain slices in hypoglycemic medium	Hypoglycemia reduces synaptic transmission. βHB increases synaptic transmission under hypoglycemic conditions. C8 does not affect synaptic transmission under hypoglycemic conditions.	(31)
Cell culture	Neuronal cell line SH-SY5Y	C8 C10 βHB	0.25 mM C8 or C10, 5 mM βHB	Cultured cells were incubated with C8, or C10, or βHB for 6 days	*C10* **Increased:** Citrate synthase activity Mitochondrial Complex I activity Catalase activity. **No change:** Mitochondrial Complex I, II, III or IV) Acyl CoA Dehydrogenase activity *βHB, C8, sebacic acid (a product of ω-oxidation of C10)* **No change:** Citrate synthase activity. C10 effect on citrate synthase is mediated through PPAR-γ	(61)
Primary cell culture	Human fibroblasts from patients with complex I deficient Leigh syndrome	C10	0.25 mM C10	6 day incubation with C10	50% of the cells responded to C10 treatment Increased: citrate synthase activity mRNA levels of PDK4, PDK3, GLYATL2, ATP5O, CPT1A, ACADVL Decreased: mRNA levels of SLC25A23, PCK2, MTHFD2, DHRS3, NDUFC1, ALDH1L2, ADHFE1 C10 effect on citrate synthase is mediated through PPAR-γ.	(63)
Primary cell culture	Cortical astrocyte culture from male CD1 mice	C8 C10	0.2 mM C8 or C10	Cultured astrocytes were incubated with C8 or C10 for 2 h	Both C8 and C10 increased basal respiration and ATP turnover. C10 increased proton leak.	(74)
Brain slices, primary cell culture	Cortical brain slices; Cultured astrocytes from male NMRI mice	C8 C10	0.2 mM C8 or C10	Isotope-labeled C8 and C10 were added to brain slices	In brain slices: C8 and C10 are actively metabolized, primarily in astrocytes. C10 is preferred over C8 as an oxidative substrate. βHB and MCFAs (C8 and C10) are metabolized in different cellular compartments. In cultured astrocytes: C8 increased ATP production, C10 increased proton leak. Glutamine generated from astrocyte C8 and C10 metabolism is utilized for neuronal GABA synthesis.	(80)
Brain slices	Brain slices from 6–16 week POMC eGFP mice	C8	0.004–0.040 mM	C8 applied *via* superfusion to the slice chamber	C8 affected the firing rates of POMC neurons: excitatory effect in some populations and inhibitory in others. C8 effect is mediated by GPR40 receptors.	(96)
Cell culture	Hypothalamic-neuron-derived N29/4 cell line	C8	0.5 mM C8	The cells were incubated with labeled C8 for up to 18 h	LCFAs are preferentially stored, while C8 is preferentially oxidized.	(96)
Brain slices	Entorhinal cortex–hippocampal slices obtained from male Sprague-Dawley rats; pentylenetetrazol (PTZ)-induced epileptiform activity	C8 C10	PTZ assay: up to 1 mM HDAC assay: up to 10 mM	C8 or C10 were applied for 40 min to the brain slices in PTZ-containing medium	C10 prevented PTZ-induced epileptiform activity and was more effective than valproic acid (VPA). C8 had no effect on epileptiform activity. C8 and C10 have no effect on Histone deacetylase activity in physiological concentrations (under 2mM).	(97)
Brain slices	Hippocampal slices obtained from Sprague-Dawley rats; PTZ-induced epileptiform activity (inhibition of GABA transmission), low Magnesium-induced epileptiform activity (potentiation of NMDA transmission)	C10 βHB	Up to 10 mM	C10 or βHB were applied for 40 min to the brain slices in PTZ-containing medium	C10 blocks epileptiform activity (induced by PTZ or low Magnesium). C10 inhibits AMPA transmission at physiological concentrations (100 μM)	(99)
Heterologous expression system	Xenopus laevis oocytes	C8 C10	Up to 5 mM	C10 or C8 were added to a preparation of oocytes engineered to express AMPA receptor subunits	C10 (but not C8) reduces AMPA transmission. C10 has stronger effect on GluA2/3 and GluA1/2 heteromeric AMPA receptors compared to GluA1 homomeric AMPA receptors. C10 binds to AMPA receptors acting as a non-competitive inhibitor. The binding site of C10 is in the channel of AMPA receptors.	(99)
Primary cell culture	Cortical astrocyte culture from 2-day-old rats	C8	0.3–0.5 mM	Cultured cells were incubated with C8 for 2 h	Astrocytes oxidize C8.	(116)
Primary cell cultures	Primary cultures established from astrocytes and oligodendrocytes obtained from 1 to 2 day-old rats, and neurons from 16 to 17 day-old rats.	C8 βHB	0.05 mM C8, 1 mM βHB	Cultured cells were incubated with labeled substrates for 3 h	Astrocytes oxidize C8 most actively, more actively than βHB or glucose. Neurons and oligodendrocytes cannot oxidize C8.	(117)
Primary cell culture	Human astrocytes obtained from adult epileptic patients undergoing neurosurgery; Astrocytes from mouse embryos.	C16	up to 200 mM	Cultured cells were incubated with labeled C16 for 2 h	Human astrocytes from adults oxidize C16. 3,3,5 triiodo-L-thyronine (T)3 stimulates fatty acid oxidation and ATP production. T3 protects astrocytes from oxidative stress and hypoglycemia. T3 neuroprotection requires fatty acid oxidation.	(118)
Cell culture	Human neuronal cell line SH-SY5Y	C8 C10	0.25 mM C8 or C10	Cultured cells were incubated with labeled C8 or C10 for 6 h	C8 and C10 were oxidized by the SH-SY5Y cells. MCFA oxidation rates were lower compared to glucose. C8 oxidation was 5-fold greater compared to C10.	(119)
Primary cell culture	Cortical astrocytes from 1 to 2 day-old rats	C8 C16	0.15 mM	Cultured cells were incubated with labeled C8 or C16 for 2 h	Ketogenesis occurred from both C8 and C16 in cultural astrocytes. Ketogenesis was more active with C8 as a substrate. Addition of △9-Tetrahydrocannabinol (THC) increased carnitine palmitoyltransferase I activity and stimulated ketogenesis from C16, but had no effect with C8 as the substrate. THC effect on ketogenesis was mediated through CB1 cannabinoid receptor but not the CB2 receptor.	(120)

*AMPA, α-amino-3-hydroxy-5-methyl-4-isoxazolepropionic acid; C10, capric acid; C8, caprylic acid; GABA, γ-aminobutyric acid; GPR40, G-protein coupled receptor 40 (also known as Free fatty acid receptor 1 (FFA1)); LCFA, long-chain fatty acids; MCFA, medium-chain fatty acids; NA, not applicable; POMC, Proopiomelanocortin; PPAR-γ, Peroxisome proliferator- activated receptor gamma; PTZ, pentylenetetrazol; βHB, β-hydroxybutyrates*.

A very insightful metabolic study has recently been conducted by Cunnane's group (39). When 20 ml of MCT were given to healthy volunteers diluted in skim milk, the ketogenic response was 2-fold stronger if the drink was given without an accompanying meal, and the C8 MCT drink was not significantly more ketogenic than the C8+C10 MCT drink. Plasma concentrations of MCFAs (measured as a fraction of total lipids without distinguishing between free and esterified forms) reached almost 0.3 mM (after consuming two 20 ml MCT drinks 4 h apart). When taken together with a meal, MCFA plasma concentrations continued to slowly increase for hours after administration. When given without a meal, C8 MCT administration resulted in a peak with a time profile similar to that of KB, however C8 and C10 plasma concentrations remained elevated without significant peaks after consumption of the C8+C10 and C10 MCT drinks. These results suggest that whatever effects C10 might directly exert in the brain, it does not matter how C10 or C8+C10 are administered. However, if one aims to maximize C8 delivery to the brain, administration of C8 triglyceride without an accompanying meal may be the best strategy.

Another factor to consider is that because most studies are designed to trigger liver ketogenesis with an implicit assumption that the higher the βHB concentration peak, the greater the effect of the intervention, MCT and various ketone supplements (salts and esters of ketone bodies) are typically given in a single dose. Several metabolic studies have demonstrated that smaller MCT doses spread out over the day may result in mild ketonemia and elevated MCFA concentrations. Courchesne-Loyer and colleagues used the following supplementation protocol to determine whether low doses of MCTs could trigger ketonemia: up to 7.5 g MCT were given to healthy volunteers 4 times a day (with meals and before bed) to a total of 30 g/day. Under these conditions, blood βHB was mildly elevated throughout the day reaching up to 0.4 mM, with the daily average increasing from about 0.1 mM pre-intervention to 0.2 mM after 4 weeks of supplementation (17). Bernini and colleagues measured blood and brain concentrations of KB and MCFAs in traumatic brain injury patients who underwent a transition from fasted state to receiving enteral nutrition, either standard or enriched with MCT (23 g/1000 Kcal) (40). There was a positive linear correlation between the blood and the brain KB concentrations. The blood and the brain KB levels decreased during the transition with no differences found between the standard nutrition vs. MCT-enriched nutrition groups. The blood MCFA increased from about 10 μM to 30-40 μM (C8 and C10) when transitioning to MCFA-enriched nutrition. Brain C8 and C10 concentrations also increased significantly. This study shows that when MCTs are mixed with other foods in low quantities and the dose is spread over rather than taken acutely, it does not elevate KB level, however it does increase the blood and brain levels of MCFAs. The effects of non-ketogenic and low-dose MCT supplementation protocols on brain functions have not yet been thoroughly investigated.

To summarize, it has been reported by different research groups in different experimental models that MCFAs exert neuroprotective effects without measurable elevation of βHB levels in the hippocampus (55, 74) or the whole brain (59, 114), while the MCFA concentrations (when they were measured) were elevated in both plasma and the brain. All the above-mentioned studies point out at the incompleteness of the paradigm prevailing in the design of human studies according to which MCT effects are entirely mediated by ketone bodies. Although it is well supported that ketogenesis from MCFA in the liver supplies the brain with alternative fuel with certain procognitive benefits, at this point, it is still an open question to what extent and which beneficial effects of MCFAs are mediated through KB and which through MCFA. A recent review by Lin and colleagues summarized available data on how to maximize the ketogenic potential of MCT ingestion, suggesting that up to 20 g of C8 triglycerides in emulsified form with coffee on empty stomach might be the best strategy (145). However, if the desired effects were mediated by MCFAs rather than KB, an entirely different strategy would be advisable, i.e. taking smaller amounts and mixing MCT with fat and carbohydrate-containing foods to increase the amount of MCFAs in peripheral circulation so that more was available for uptake by the brain.

At this point, there is currently no agreement across the studies on the optimal target therapeutic concentrations of either βHB or MCFAs. Since βHB or MCFAs are involved in multiple metabolic and signaling pathways, and since the effects of MCT supplementation seem to be brain-region- and phenotype-specific, it is possible that different concentrations may be required to achieve different effects in different models and different brain regions. More detailed animal studies are required to better understand the complex effects of MCT metabolites in the brain.

## Metabolic Effects of MCT Supplementation

Over the past several decades, a large number of studies have looked at the metabolic effects of MCT consumption either in the form of a high-fat diet or when using MCFAs as a substitute for dietary LCFAs. Although the overall conclusion is that MCFAs in large amounts are far less hepatotoxic than large amounts of LCFAs, one can find many conflicting results. For instance, a MCFA-rich diet compared to a LCFA-rich diet increased (137), decreased (138), or had no effect (139) on TG accumulation in the liver; increased (41, 42, 146, 147), decreased (137, 140, 141), or had no effect (43) on fasting plasma TG concentration; increased (41–43), decreased (44), or had no effect (45) on fasting plasma total cholesterol concentrations (Table 2). The reader may be referred to a recent meta-analysis of differential effects of medium- and long-chain saturated fatty acids on the blood lipid profile (148).

According to a review of toxicological properties of MCT, 1 g/kg dose has been confirmed as safe (19). However, when MCTs are taken for their ketogenic properties, it is reasonable to try and administer a higher dose to achieve higher blood levels of ketone bodies. In one case report, seizures were significantly reduced in a 43-year-old man after supplementing his regular diet with four tablespoons of MCT twice daily (which adds up to about 112 g/day) (47). Large oral doses of MCT may cause osmotic diarrhea, especially when taken on an empty stomach. However, the tolerance is better when taken with meals (27). Since MCFAs, LCFAs, and glucose participate in many intertwined metabolic pathways, one must be cautious when taking increasingly high amounts of MCT without any dietary restrictions. While obesity, insulin resistance, and diabetes are common risk factors for developing AD, all sharing dyslipidemia as a pathological mechanism (149), human clinical trials of MCT effects on cognitive functions generally do not include participants with metabolic disorders (20, 21, 29, 46).

The studies of MCT supplementation of regular diet on markers of metabolic health are still limited. A recent clinical study in patients with MCI receiving 30 g of MCT for 6 weeks assessed a range of cardiometabolic and inflammatory markers and only found a 2.5-fold elevation of IL-8 (46). In one animal study, supplementation of standard feed with MCT in juvenile male rats (10 g/kg) allowed to achieve plasma βHB levels of up to 5 mM for almost 8 h with a parallel almost-2-fold decrease in plasma glucose (142). However, when administered in such a high dose, after 28 days, the liver mass significantly increased while the HDL cholesterol significantly decreased, which raises a concern that MCT added in high amounts to a standard diet without dietary restrictions can be hepatotoxic. It must be noted, however, that, taking into account the rat metabolic rate, the administered dose of 10 g/kg MCT is only 2–5 times higher than the doses used in human clinical trials. In a human study, when a total of 20–30 g of MCT were given in four separate 5–7.5 g doses, no changes were observed in plasma TG and cholesterol concentrations (142). The effects of MCT supplementation will likely be highly variable depending on the constituents of the diet, the timing of meals, and the absence or presence of underlying metabolic disorders. At this moment, the long-term effects of MCT supplementation on cardiovascular risk factors and glucose metabolism are unknown.

It should also be noted that although MCT are ketogenic in the presence of carbohydrates and taking MCT with meals is known to improve gastrointestinal tolerability, administering large MCT doses together with carbohydrates is not advisable. Insulin activates acetyl CoA-carboxylase (150), which increases the cellular concentration of malonyl-CoA, which in turn results in more acetyl-CoA redirected toward *de novo* lipogenesis, less oxidation of LCFAs and more LCFAs available for esterification to form TG and cholesterol esters. Therefore, to maximize the ketogenic effect of MCT administration and at the same time to minimize the metabolic consequences of taking fats and carbohydrates together in excess, it may be advisable to take MCT after an overnight fast. Alternatively, administering MCT in several small doses spread out throughout the day may also reduce the likelihood of adverse effects in the liver. As mentioned, very few studies to date investigated the neuroprotective effects of this approach (Table 1).

## Future Perspectives and Conclusions

The supplementation of a regular diet with MCT is a promising approach to improve cognitive functions in healthy individuals and those suffering from age- or disease-related cognitive impairment. The effects of MCT supplementation on cognitive functions seem to be both phenotype-specific and brain-region-specific. It appears that both ketone bodies and MCFAs directly mediate these effects *via* partially overlapping and interacting pathways. More research is needed to better understand the underlying mechanisms of these effects. Ideally, more studies should implement experimental designs allowing to distinguish between the effects of βHB and the two MCFAs. For example, chronic MCFA injections into carotid artery could be used to study chronic effects of MCFAs *in vivo*. Since most studies were conducted with the assumption that the neuroprotective effects of MCT depend on ketogenesis, single doses and larger concentrations were used. Several existing studies point at the possibility that several small doses throughout the day may also offer certain cognition-enhancing benefits. This approach requires further study.

Despite a large amount of research on the effects of MCT ingestion on metabolic health, the consequences of MCT supplementation of a regular diet for the purpose of improving cognition have not been sufficiently investigated, especially when taken at higher doses without reduction of carbohydrate intake. With the increasing popularity of MCT supplementation and growing public awareness of its potential cognition-enhancing effects, more research is needed to clarify the long-term effects of MCT supplementation on cardiovascular and metabolic health, as well as how MCTs are being used outside clinical settings.

## Author Contributions

KS and AT conceptualized the article and wrote the original draft. KS prepared the figures. KS, AT, AS, MK, and SA contributed to reviewing and editing of the manuscript. All authors contributed to the article and approved the submitted version.

## Funding

This work was supported by Russian Science Foundation, project No. 19-75-10076.

## Conflict of Interest

The authors declare that the research was conducted in the absence of any commercial or financial relationships that could be construed as a potential conflict of interest.

## Publisher's Note

All claims expressed in this article are solely those of the authors and do not necessarily represent those of their affiliated organizations, or those of the publisher, the editors and the reviewers. Any product that may be evaluated in this article, or claim that may be made by its manufacturer, is not guaranteed or endorsed by the publisher.
